# Quantitative model for imaging single fiber reflectance spectroscopy

**DOI:** 10.1038/s41598-026-48855-y

**Published:** 2026-04-14

**Authors:** Robin van Zutphen, Ton G. van Leeuwen, Xavier Attendu

**Affiliations:** 1 Biomedical Engineering and Physics, Amsterdam UMC location University of Amsterdam, Meibergdreef 9, Amsterdam, The Netherlands; 2 Imaging and Biomarkers, Cancer Center Amsterdam, Amsterdam, The Netherlands; 3 Heart failure & arrhythmias, Amsterdam Cardiovascular Sciences, Amsterdam, The Netherlands

**Keywords:** Imaging single fiber reflectance spectroscopy, Monte Carlo photon transport, Optical properties, Optics and photonics, Physics

## Abstract

Single-fiber reflectance (SFR) spectroscopy enables quantitative retrieval of tissue optical properties from highly localized measurements through a single multimode fiber, but its diagnostic yield can be limited by small sampling volumes and probe-pressure artifacts. Imaging SFR (iSFR) mitigates these issues by enabling contact-free, point-scanning spectral imaging using optics to project the fiber’s illumination–collection cone onto the tissue surface. We present a quantitative model tailored to iSFR that supports inverse retrieval of tissue absorption and scattering properties from reflectance spectra. The model is based on large-scale Monte Carlo simulations using a generalizable reflectance computation that replaces explicit simulation of arbitrary finite source–detector configurations with a distance-based probability weighting, substantially reducing computational cost. Across broad optical and geometric ranges, the iSFR model predicts reflectance with a median error of 6.2% (SFR: 4.2%). In inverse mode, using wavelength parameterizations for optical properties, the approach recovered absorption and scattering coefficients to within approximately 10% over a wide range of added noise for simulated spectra from two simplified tissue models, serving as a proof-of-concept. These results establish a quantitative model and an efficient computational pipeline for (i)SFR spectroscopy.

## Introduction

Reflectance spectroscopy (RS) is a widely used optical technique for probing matter by analyzing the spectrum of light reflected after broadband illumination^1^. As photons propagate through the medium, scattering and absorption events imprint wavelength-dependent features on the reflected spectrum, providing signatures of the underlying optical properties. In biomedical applications, analyzing these signatures enables non-invasive tissue characterization and has shown promise for early cancer detection across multiple studies^2,3^. Beyond medical tissue diagnostics, RS and related techniques such as single-fiber reflectance (SFR), diffuse reflectance spectroscopy (DRS), and hyperspectral imaging (HSI) have been applied in diverse contexts, including agriculture^4,5^, forensics^6^, and materials science^7^. Analyses may rely on relative spectral comparisons between sites or on quantitative extraction of optical properties, the latter being the central objective of this work. Quantitative approaches provide direct access to absorption and scattering parameters, allowing comparisons across systems and experimental conditions and yielding physically interpretable sample metrics. When considering biological samples, RS is sensitive to microstructural and biochemical alterations (e.g., epithelial thickening, nuclear pleomorphism, altered glandular architecture, neovascularization) that often arise before visible morphological changes. Such early features escape detection by conventional imaging. Consequently, RS offers a non-invasive and quantitative route to early cancer detection, providing a strong complementary or alternative approach to tissue biopsy.

SFR uses a single multimode fiber for both illumination and detection, resulting in compact and flexible probes well suited for in vivo and minimally invasive measurements. SFR has shown clinical promise in a wide range of applications, including the detection of cervical premalignancies^8^, metastatic lymph nodes^9^, lung cancer^10^, pancreatic cancer^11^, brain tissue oxygenation^12^, laryngeal cancer^13^, and esophageal neoplasia in Barrett’s esophagus^14^. However, because SFR relies on a single fiber that must be physically positioned against the tissue at each site, it remains limited to point-based measurements. Consequently, spatial coverage is restricted, which reduces diagnostic yield. In addition, probe contact can introduce variability through pressure-induced changes in local optical properties, which compromise accuracy and reproducibility^15,16^.

**Fig. 1:**
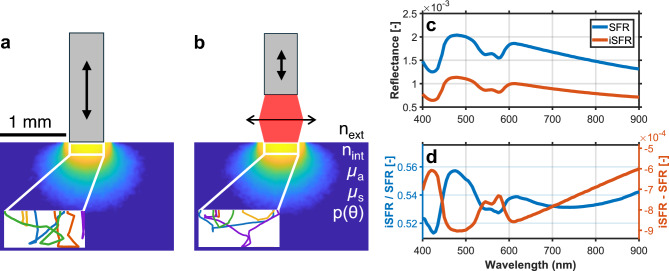
Monte Carlo simulations illustrating the probing volumes and representative detected photon trajectories for SFR (**a**) and iSFR (**b**) using the same fiber (core diameter 500 µm, NA = 0.22) and identical biologically relevant optical properties. Panel (**b**) is intended as a schematic representation of an optical path/focusing configuration that projects the fiber onto the tissue surface, rather than a bare fiber held directly above the tissue; for actual example implementations, see:^17–22^. The photon trajectories show that scattering directions are not fully randomized in these modalities, highlighting the importance of phase function dependent transport, as discussed in the Theory section. The corresponding reflectance spectra (**c**) appear as scaled versions of each other; however, the ratio and the difference between the spectra (**d**) deviate from linear scaling. Consequently, the conventional SFR model, even under matched NA and fiber size conditions, introduces additional errors that cannot be readily corrected by simple scaling.

Imaging single-fiber reflectance spectroscopy (iSFR) extends SFR to a non-contact configuration, in which illumination and collection occur through fibers that are optically imaged onto the tissue surface. This configuration enables point-scanning spectral imaging over larger tissue areas while preserving endoscopic integratability. Although SFR and iSFR differ substantially in how stable acquisition is achieved, their modeling requirements are closely related. When the goal is to compute reflectance at the tissue surface for a given illumination and collection geometry, the differences reduce to two key aspects: (i) iSFR introduces an air–tissue rather than glass–tissue interface, increasing the influence of boundary effects, and (ii) the illumination and collection spot size and numerical aperture (NA) are set by the optical imaging configuration rather than by intrinsic fiber properties, permitting a different range of achievable values. As a result, established SFR models do not automatically carry over to iSFR configurations, and updated model parameters are required to ensure quantitative accuracy. Figure 1 schematically illustrates the two modalities by showing the probing volumes of SFR and iSFR for identical NAs and fiber or spot sizes. For a representative set of skin-like optical properties, the corresponding reflectance spectra, obtained from Monte Carlo simulations, are shown for both modalities. Despite identical measurement geometries and nearly indistinguishable spectral shapes, neither the ratio nor the difference between the two spectra follows a simple linear scaling, highlighting the need for an updated model to accurately describe iSFR reflectance.

Several groups have introduced fiber-based imaging approaches in which multispectral or hyperspectral illumination and collection are optically imaged onto the tissue surface using reflective or refractive optics^17–22^. While these studies demonstrate the feasibility of non-contact, fiber-based spectral imaging and multimodal integration, quantitative estimation of tissue optical properties from the acquired reflectance data was not addressed due to the lack of appropriate models. In many of these implementations, iSFR or related single-fiber imaging methods are integrated with optical coherence tomography (OCT), often using double-clad fibers. In such configurations, iSFR shares the OCT optical path and therefore operates at NAs far below those typically used in conventional SFR, as OCT relies on low NAs ($\sim$ 0.01–0.03) to maintain a large depth of field. Consequently, a quantitative reflectance model is needed that remains accurate at low numerical apertures, motivating the development of an updated model explicitly validated across an extended NA range.

The goal of this work is to develop a quantitative model for extracting tissue optical properties from reflectance spectra acquired with iSFR. To this end, we first established a computational pipeline for efficiently calculating reflectance across arbitrary source–detector geometries using large-scale Monte Carlo (MC) simulations. Second, this pipeline was benchmarked against the established SFR model by Post *et al.*^23,24^, incorporating several corrections and algorithmic improvements into the original framework to ensure consistency and accuracy. Third, building on this validated foundation, we constructed a model tailored to iSFR configurations, capable of accurately retrieving optical properties from simulated reflectance spectra, including those acquired in low-NA regimes. The resulting framework provides quantitative and accurate models for both contact (SFR) and non-contact (iSFR) fiber-based reflectance spectroscopy, establishing a basis for experimental validation and future clinical translation.

## Theory

### Subdiffuse reflectance

Photon transport in tissue is governed by three optical properties: the absorption coefficient ($\mu _a$), the scattering coefficient ($\mu _s$), and the scattering phase function $p(\theta )$. Together, these parameters determine the transport of light through the illuminated tissue and ultimately the reflected light measured at the surface. Absorption attenuates the photon weight along the optical path according to the Beer–Lambert law. Because detected photons traverse a distribution of path lengths $p(l)$, the measured intensity can be expressed as

1 $$\begin{aligned} I = I_0 \int _0^{\infty } p(l)\, e^{-\mu _a l}\, dl, \end{aligned}$$

where $p(l)$ represents the probability density of photon path lengths contributing to the detected signal. Scattering is quantified by the scattering coefficient $\mu _s$ [mm$^{-1}$], and by the scattering phase function $p(\theta )\,[\textrm{sr}^{-1}]$, which specifies the probability density for deflection about the polar angle $\theta$. A common metric to describe the combined effect of $\mu _s$ and $p(\theta )$ is the reduced scattering coefficient,

2 $$\begin{aligned} \mu _s' = \mu _s (1 - g), \end{aligned}$$

where the anisotropy factor $g$ is defined as the first angular moment of the phase function,

3 $$\begin{aligned} g=\langle \cos \theta \rangle =2\pi \int _0^\pi \cos \theta \,p(\theta )\,\sin \theta \,d\theta . \end{aligned}$$

To relate these optical properties to the measured reflectance, the most commonly used framework is diffusion theory, a first-order approximation to the radiative transfer equation. However, due to the overlapping source–detector geometry in (i)SFR and the typical range of tissue scattering coefficients, most photons undergo only a few scattering events before detection. As a result, the assumptions underlying the diffusion approximation are no longer valid, as photons have not scattered sufficiently to randomize their propagation directions and be considered *diffuse*^25,26^. In this *subdiffuse* regime, the reflectance depends on the detailed form of the phase function $p(\theta )$, while the mean cosine $g$, used in the diffusion approximation, is no longer an adequate descriptor of photon transport^27–29^.

Deriving closed-form analytical solutions in this *subdiffuse* regime is non-trivial, and although several models have been proposed to approximate subdiffuse reflectance or to extend the range of validity of the diffusion approximation^30–35^, these approaches typically remain incomplete. While some achieve reasonable quantitative accuracy, challenges regarding phase-function parametrizations and inversion, as well as overlapping source–detector geometries, are not fully addressed. Along similar lines, recent work by Wilk *et al.* extended classical diffusion theory by accounting for the finite speed of light, providing a framework that incorporates both subdiffuse and diffuse transport while avoiding unphysical near-source behavior^36^. However, this approach cannot explicitly incorporate user-defined phase functions and still relies on finite-element modeling of specific acquisition geometries to retrieve optical properties. Consequently, MC simulations remain the most accurate and widely used method for describing subdiffuse reflectance. Among semi-empirical models derived from MC simulations^37–39^, the SFR model by Post *et al.*^23,24^ provides the highest quantitative accuracy to date. It therefore forms the foundation for the present work, where it is adapted and extended to iSFR configurations.

### Phase function parameterization

To accurately describe reflectance in the subdiffuse regime, several studies have sought improved parameterizations that capture the angular dependence of scattering beyond the parameter $g$, such as $\gamma$^40^, $\delta$^41^, and $\sigma$^28^, which are derived using higher-order moments of the phase function. While these improve predictive performance for SFR reflectance compared to using $g$ alone, significant discrepancies can persist when considering the full range of phase functions encountered in biological tissues^23^. Post *et al.*^23^ proposed an alternative descriptor based on their definition of semi-ballistic transport as multiple forward scatterings followed by a single backward scattering. They showed that this probability can be captured by

4 $$\begin{aligned} p_{sb} = \frac{p_{\text {back}}}{1 - p_{\text {forward}}}, \end{aligned}$$

where $p_{\text {forward}}$ and $p_{\text {back}}$ are the probabilities of scattering into predefined forward and backward angular ranges, obtained by integrating the normalized phase function $p(\theta )$. By simulating a highly subdiffuse, absorption-free SFR configuration and fixing other parameters that influence the reflectance, they isolated the effect of the phase function and its parametrization $p_{sb}$ on the reflectance. The angular integration limits defining $p_{sb}$ were chosen to minimize the relative dispersion of the phase-function descriptor for a given reflectance. By ensuring that phase functions yielding identical reflectance map to similar values of $p_{sb}$, the parametrization reduces phase-function–induced degeneracy and improves conditioning of the inverse problem. The resulting definition that minimized this spread is

5 $$\begin{aligned} p_{sb}=\frac{p_b(1^\circ )}{1-p_f(23^\circ )} =\frac{\displaystyle 2\pi \int _{179^\circ }^{180^\circ } p(\theta )\,\sin \theta \,\textrm{d}\theta }{\displaystyle 1-2\pi \int _{0^\circ }^{23^\circ } p(\theta )\,\sin \theta \,\textrm{d}\theta }. \end{aligned}$$

Since it was previously found that the optimal integration angles depend weakly on NA, and iSFR can be performed over a wider range of NAs than conventional SFR, we verified the validity of $p_{sb}$ for iSFR geometries. These results are presented in the Supplementary Section 1.

### Ratio of diffuse and subdiffuse contributions

Rather than modeling semi-ballistic reflectance directly, Post *et al.*^23,24^ expressed the total reflectance ($R_{\text {SFR, total}}$) as the sum of a diffuse and a subdiffuse component,

6 $$\begin{aligned} R_{\text {SFR, total}} = (1 + X)\, R_{\text {SFR, diffuse}}, \end{aligned}$$

where $X$ represents the ratio between subdiffuse and diffuse contributions:

7 $$\begin{aligned} X = \frac{R_{\text {SFR, subdiffuse}}}{R_{\text {SFR, diffuse}}} = a_2 \cdot \left( \frac{p_{sb}}{(\mu _s' \cdot d_f)^2} \right) ^{a_3} \cdot \exp \!\left[ b_1 \left( \frac{\mu _a}{\mu _s'} \right) ^{b_2} \right] . \end{aligned}$$

This formulation captures how the total reflectance depends on the ratio between diffuse and subdiffuse photon contributions as a function of geometric and optical parameters. The diffuse term $R_{\text {SFR, diffuse}}$ is obtained by integrating the radial diffuse reflectance $R_{\text {diffuse}}(\rho ,\mu _a,\mu _s')$ over all distances $\rho$ between two points on the circular detection area of fiber diameter $d_f$, as derived by Faber *et al.*^42^:

8 $$\begin{aligned} R_{\text {SFR, diffuse}} = \frac{\pi }{4}\, d_f^2 \int _0^{d_f} R_{\text {diffuse}}(\rho ,\mu _a,\mu _s')\, p(\rho ,d_f)\, d\rho , \end{aligned}$$

where $p(\rho , d_f)$ describes the probability distribution of distances between two random points on the disk:

9 $$\begin{aligned} p(\rho ,d_f)=\frac{16\rho }{\pi d_f^{2}}\cos ^{-1}\!\left( \frac{\rho }{d_f}\right) -\frac{16}{\pi d_f}\left( \frac{\rho }{d_f}\right) ^{2}\sqrt{1-\left( \frac{\rho }{d_f}\right) ^{2}} \end{aligned}$$

and $R_{\text {diffuse}}(\rho ,\mu _a,\mu _s')$ denotes the diffuse reflectance at radial distance $\rho$ from a pencil-beam source using the extrapolated boundary condition^43^:

10 $$\begin{aligned} R_{\text {diffuse}}(\rho ,\mu _a,\mu _s') = \frac{a'}{4\pi } \left[ z_0\!\left( \mu _{\text {eff}} + \frac{1}{r_1}\right) \frac{e^{-\mu _{\text {eff}} r_1}}{r_1^{2}} + (z_0 + 2 z_b)\!\left( \mu _{\text {eff}} + \frac{1}{r_2}\right) \frac{e^{-\mu _{\text {eff}} r_2}}{r_2^{2}} \right] , \end{aligned}$$

with

$$a' = \frac{\mu _s'}{\mu _s' + \mu _a},\qquad z_0 = \frac{1}{\mu _s'},\qquad \mu _{\text {eff}} = \sqrt{3\,\mu _a\,\mu _s'},$$

$$r_1 = \sqrt{z_0^{2} + \rho ^{2}},\qquad r_2 = \sqrt{(z_0 + 2 z_b)^{2} + \rho ^{2}},\qquad z_b = \frac{2A}{3\,\mu _s'}.$$

Boundary effects are included through $z_b$, where $A$ depends on the refractive index mismatch: $A = 1.027$ for a glass–tissue interface ($n = 1.45/1.35$) and $A = 2.6355$ for an air–tissue interface ($n = 1.00/1.35$)^44^. Assuming Lambertian angular emission, the angular collection efficiency is limited by the fiber NA:

11 $$\begin{aligned} \eta _c = \left( \frac{\text {NA}}{n_{\text {tissue}}} \right) ^2, \end{aligned}$$

with an empirical factor $a_1$ introduced to account for deviations from the ideal Lambertian assumption.

12 $$\begin{aligned} R_{\text {SFR, diffuse}} \;\longrightarrow \; \eta _c \cdot a_1 \cdot R_{\text {SFR, diffuse}}. \end{aligned}$$

By fitting Eqs. (6)–(12) to large Monte Carlo datasets spanning a wide range of phase functions, optical properties, and fiber geometries, Post *et al.* determined the empirical parameters $a_1, a_2, a_3, b_1,$ and $b_2$ for different NAs (0.1, 0.22, 0.50). In this work, we utilized the same methodology but applied it to iSFR geometries and boundary conditions.

## Methods

### Parameter space

To determine the optimal values of the model parameters $a_1$, $a_2$, $a_3$, $b_1$, and $b_2$, we fitted the reflectance model [Eqs. (6)–(12)] to a large dataset of Monte Carlo-simulated reflectance values, covering a broad range of optical and geometrical properties (Tables 1 and 2). For the geometrical parameters, we considered typical fiber diameters and NAs of commercially available multimode fibers. In conventional SFR, the spot size corresponds to the physical diameter of the fiber core, whereas in iSFR it is defined by the projected illumination spot on the sample surface. Because iSFR relies on free-space collimation and focusing rather than physical fiber contact, the achievable spot size can be adjusted more flexibly. The explored parameter space therefore extends beyond that of standard SFR, covering a wider range of spot sizes and NAs. For iSFR, we assumed a magnification below unity, corresponding to NAs of 0.22 and lower. Optical property ranges were selected to represent values commonly reported for biological tissues under visible and near-infrared illumination^45^. For the scattering phase functions, we followed the analysis of Witteveen *et al.*^46^, who showed that the two-term Henyey–Greenstein (TTHG), modified Henyey–Greenstein (MHG), and Reynolds–McCormick (RMC) functions most accurately reproduce experimentally measured tissue phase functions. All three models were therefore included. From the full phase-function grid (Table 2), we excluded combinations with $g_1 > 0.95$ or $g_2 < 0.5$, following the filtering criterion introduced by Post *et al.*^23^. Here, $g_1$ and $g_2$ denote the first and second Legendre moments of the phase function^40^, respectively, with $g_1$ corresponding to the conventional anisotropy factor $g$ defined in Eq. (3). After filtering, the final dataset consisted of 146 TTHG, 15 MHG, and 46 RMC phase functions. These selections are nearly identical to those used in the original SFR model^23^ and were shown to roughly match experimentally measured tissue phase-function distributions^46^. For the included phase functions, the derived $p_{sb}$ values formed an approximately log-normal distribution spanning $10^{-6}$ to $10^{-4}$. Combined with the corresponding anisotropy factors $g$ and reduced scattering coefficients $\mu _s'$, this resulted in scattering coefficients $\mu _s$ ranging from 1 to 400 mm$^{-1}$. To test whether the assumptions underlying $p_{sb}$ remain valid for iSFR, we repeated the original derivation analysis, as detailed in Supplementary Section 1, and found that $p_{sb}$ remains an accurate parameterization of the tissue phase function, with no changes to the original formulation required^23^.

**Table 1:** Parameter ranges for geometrical and optical properties.

Parameter	Values
System geometries	
NA	SFR: 0.10, 0.22, 0.39, 0.50
	iSFR: 0.01, 0.025, 0.05, 0.10, 0.22
Spot size	SFR: 0.1–1.0 mm (0.1 mm steps)
	iSFR: 0.2–2.0 mm (0.2 mm steps), 2.5 mm
Optical properties	
Refractive index, medium	$n = 1.35$
Refractive index, external	SFR: 1.45 iSFR: 1.00
Reduced scattering coefficient	0.5, 1, 2.5, 5, 7.5, 10, 15, 20 mm$^{-1}$
Absorption coefficient	0.01, 0.025, 0.05, 0.075, 0.1, 0.25, 0.5, 0.75, 1, 2.5, 5, 7.5, 10 mm$^{-1}$
Phase functions	146 TTHG, 15 MHG, 46 RMC (see Table 2)

**Table 2:** Parameter ranges and analytical definitions of the included phase functions.

Model	Parameters	Formula
MHG^47^	$0.01 \le g_{\textrm{HG}} \le 0.95$ (10 steps)	$\displaystyle p(\theta ) = \alpha \,\frac{1-g_{\textrm{HG}}^2}{4\pi \,(1+g_{\textrm{HG}}^2-2g_{\textrm{HG}}\cos \theta )^{3/2}} + (1-\alpha )\,\frac{3\cos ^2\theta }{4\pi }$
	$$0.01 \le \alpha \le 0.99$$ (10 steps)	
TTHG^48^	$0.5 \le \alpha \le 0.9$ (3 steps)	$\displaystyle p(\theta ) = \alpha \,\frac{1-g_{\mathrm f}^2}{4\pi \,(1+g_{\mathrm f}^2-2g_{\mathrm f}\cos \theta )^{3/2}} + (1-\alpha )\,\frac{1-g_{\mathrm b}^2}{4\pi \,(1+g_{\mathrm b}^2-2g_{\mathrm b}\cos \theta )^{3/2}}$
	$$0.91 \le \alpha \le 0.99$$ (5 steps)	
	$$0.05 \le g_{\mathrm f} \le 0.95$$ (10 steps)	
	$$-0.95 \le g_{\mathrm b} \le -0.05$$ (5 steps)	
RMC^49^	$0.01 \le \alpha \le 2.5$ (10 steps)	$$\begin{aligned} p(\theta )&= K\,(1+g_{\mathrm R}^2-2g_{\mathrm R}\cos \theta )^{-(\alpha +1)}, \\ K&= \frac{\alpha g_{\mathrm R}}{\pi }\,(1-g_{\mathrm R}^2)^{2\alpha } \left[ (1+g_{\mathrm R})^{2\alpha }-(1-g_{\mathrm R})^{2\alpha }\right] ^{-1} \end{aligned}$$
	$$0.01 \le g_{\mathrm R} \le 0.95 - 0.2\alpha$$ (10 steps)	

### Monte Carlo algorithm

To efficiently generate the reflectance dataset across all parameter combinations in Tables 1 and 2, we employed two main strategies to minimize the number of computationally intensive MC runs. First, we used the GPU-accelerated, voxel-based MC engine MCX (Monte Carlo eXtreme)^50^, specifically its MATLAB interface MCXLAB, using MCX v2025.10. The software was executed in MATLAB R2025a on a Linux workstation running Ubuntu 24.04.2 LTS (Noble Numbat). MCX implements a microscopic Beer–Lambert law ($\mu$-BLL) approach, in which photon trajectories are sampled based solely on the scattering properties of the medium. This allows all simulations to be performed without absorption. By storing the path lengths of detected photons, absorption can subsequently be applied retrospectively through exponential attenuation (Eq. 1), enabling reflectance to be computed for arbitrary absorption coefficients in post-processing.

Second, we extended the approach of Faber *et al.*^42^, who showed that within the diffusion-theory framework and under certain assumptions, the reflectance depends solely on the radial distance between photon entry and exit points on the fiber face, rather than on their absolute coordinates. While this property was previously used only to compute the diffuse contribution (Eq. 12), here we apply the same single-integral approximation (SIA) to the *total* reflectance obtained from MC simulations. The concept of treating the response to a pencil beam or point source as a spatial impulse and extending it to broader illumination profiles has a long history in optical Monte Carlo modeling^51,52^. In this work, we generalize the SIA principle beyond the diffusion regime and use it to compute reflectance for arbitrary source–detector geometries based solely on photon exit coordinates and path lengths. This approach eliminates the need for fluence calculations and substantially improves computational efficiency. Specifically, we simulate reflectance as a function of radial exit distance using MC and subsequently obtain the total reflectance for any fiber diameter by integrating this output with the analytical distance distribution between two random points on a disk, analogous to Eq. 12. A more detailed implementation and statistical validation are provided in Supplementary Section 2.1.

Combining the $\mu$-BLL and SIA approaches effectively removes two dimensions from the parameter space that must be directly sampled by MC simulations. Instead of computing separate runs for each combination of $\mu _a$ and $d_f$, we performed a reduced set of simulations spanning only $\mu _s'$, NA, and phase function, from which reflectance for arbitrary absorption and fiber diameter values were computed in post-processing. Simulations were performed in a homogeneous, semi-infinite medium of size $10 \times 10 \times 10~\textrm{cm}^3$ to eliminate boundary effects. The domain was represented by a single voxel to suppress unnecessary fluence calculations, and spatial scaling of photon coordinates and path lengths was applied accordingly. Photons were launched in batches of $5 \times 10^7$ from a point source at the center of the volume, using a uniform angular distribution matched to the NA and corrected for the refractive index of the medium. For conventional SFR, the launch NA corresponds directly to the numerical aperture of the fiber. For iSFR, the launch NA was taken as the effective NA of the projected beam at the sample surface. The entire launch plane was treated as the detector surface. Fresnel reflections were applied only for photons exiting the medium (tissue to external), since source-side Fresnel effects are accounted for experimentally through calibration^53^. Photons exiting at angles exceeding the corresponding NA or at radial positions larger than the maximum simulated spot size ($1~\textrm{mm}$ for SFR and $2.5~\textrm{mm}$ for iSFR) were discarded. The resulting photon data were used to construct $R(\rho )$, the reflectance as a function of radial exit distance, from which the total reflectance $R_{\textrm{tot}}$ for any fiber size was obtained by numerical integration. Each simulation was repeated until a sufficient number of photons were detected to ensure that the stochastic noise on the computed reflectance was below 1%. A complete overview of the simulation and post-processing pipeline, including additional measures taken to ensure robustness, accuracy, and statistical reliability, is provided in Supplementary Section 2, and an example MATLAB script is published to demonstrate the core concepts and MC implementation.

### Model derivation

The final dataset comprised all parameter combinations listed in Tables 1 and 2, totaling approximately $1.1 \times 10^6$ parameter combinations for iSFR and $0.9 \times 10^6$ for SFR. These data were fitted using Eqs. (6)–(12) to determine the model parameters $a_1$, $a_2$, $a_3$, $b_1$, and $b_2$. Fitting was performed using nonlinear least-squares optimization, with a relative least-squares objective function. This weighting prevents bias toward larger reflectance values, which span several orders of magnitude across the dataset. We employed a two-step fitting procedure in which, in the first step, we optimized the scattering-related parameters $a_1$, $a_2$, and $a_3$ using only the absorption-free subset of the data ($\mu _a = 0$):

13 $$\begin{aligned} \{ \hat{a}_1, \hat{a}_2, \hat{a}_3 \} = \underset{\{a_1, a_2, a_3\}}{\mathrm {arg\,min}} \; \sum _{\begin{array}{c} i=1 \\ \mu _{a,i}=0 \end{array}}^{N} \left[ \frac{ R_{\textrm{model},i}(a_1,a_2,a_3) - R_{\textrm{MC},i} }{ R_{\textrm{MC},i} } \right] ^2 . \end{aligned}$$

In the second step, we refined the absorption-related parameters $b_1$ and $b_2$ using the full dataset, while keeping $a_1$, $a_2$, and $a_3$ fixed at their previously obtained values $\hat{a}_1$, $\hat{a}_2$, and $\hat{a}_3$:

14 $$\begin{aligned} \{ \hat{b}_1, \hat{b}_2 \} = \underset{\{b_1, b_2\}}{\mathrm {arg\,min}} \; \sum _{i=1}^{N} \left[ \frac{ R_{\textrm{model},i}(\hat{a}_1,\hat{a}_2,\hat{a}_3,b_1,b_2) - R_{\textrm{MC},i} }{ R_{\textrm{MC},i} } \right] ^2 . \end{aligned}$$

This sequential approach isolates the estimation of scattering-related parameters from additional inaccuracies introduced by the exponential absorption term (discussed in the main Discussion and in the Supplementary Materials). The only constraint imposed during fitting was that all parameters remain positive. Approximate 95% confidence intervals for the fitted parameters were computed in MATLAB using nlparci, based on the residuals and Jacobian of the nonlinear least-squares fit; these intervals reflect local parameter uncertainty under the adopted model formulation and fit methodology only.

### Inverse parameter retrieval

The developed model is intended for inverse-problem settings, in which measured reflectance spectra are used to estimate underlying optical properties for a known acquisition geometry (spot diameter $d_f$ and NA). To demonstrate its performance, we generated reflectance spectra using MC simulations for two representative tissue types, skin and generic soft tissue, using a spot size of $500~\mu \textrm{m}$ and $\textrm{NA} = 0.05$. Spectra were simulated between 400 and 900 nm in 5 nm steps. To ensure an overdetermined inverse problem (i.e., more spectral measurements than variables), the wavelength dependence of the optical properties was described using the following parameterizations as a function of wavelength. The reduced scattering coefficient $\mu _s'(\lambda )$ was modeled using a power law^45^, the absorption coefficient $\mu _a(\lambda )$ as a linear combination of oxygenated and deoxygenated hemoglobin spectra^54^ weighted by the blood volume fraction $v_{f,\text {blood}}$, with the relative contributions determined by the oxygen saturation $sO_2$, and the phase-function descriptor $p_{sb}(\lambda )$ as a third-order polynomial without direct physical constraints^55^. The wavelength-dependent parameterizations of $\mu _s'(\lambda )$ and $p_{sb}(\lambda )$ are expressed relative to a reference wavelength $\lambda ^* = 650~\textrm{nm}$. These parameterizations, together with reference parameter values for skin and soft tissue^45^, are summarized in Table 3. Because MC simulations require a complete phase function rather than a scalar $p_{sb}$, we adopted a two-term Henyey–Greenstein (TTHG) function, with parameters chosen such that the resulting $p_{sb}$ values lay near the center of the biologically relevant range and the anisotropy decreased linearly with wavelength. This phase-function was chosen arbitrarily, as there is no consensus on which phase functions best describe specific tissue types.

To mimic experimental variability, Gaussian noise corresponding to 0.1–5% of the reflectance root-mean-square (computed over the full wavelength range) was independently added to each spectral point, yielding 250 random noisy reflectance spectra per tissue type and per noise level. The underlying ground-truth spectra were generated using the described MC approach with minimized stochastic noise by simulating at least $10^6$ detected photons per wavelength. This photon count ensures that residual MC noise remains negligible compared to the imposed noise levels across the investigated parameter range (see Supplementary Section 2.5).

Optical property retrieval was then performed via nonlinear least-squares fitting using a relative least-squares objective function. The optimization problem was defined as

15 $$\begin{aligned} \underset{\theta }{\mathrm {arg\,min}} \sum _{\lambda } \left[ \frac{ R_{\textrm{model}}(\lambda ; \theta ) - R_{\textrm{MC}}(\lambda ) }{ R_{\textrm{MC}}(\lambda ) } \right] ^2, \end{aligned}$$

where $\theta = \{a, b, v_{f,\text {blood}}, sO_2, p_1, p_2, p_3\}$ denotes the set of fitted parameters defined in Table 3. To improve robustness against local minima, we employed a multistart strategy with 50 randomized initializations within the parameter bounds. This procedure was applied to all 250 noisy spectra at each noise level, providing a first indication of the model’s inverse-retrieval performance under noisy conditions. Further details on solver settings, parameter bounds, and convergence criteria are provided in the Supplementary Section 5.

Although more complex absorption and noise models, incorporating additional chromophores or physiological parameters such as apparent vessel size, could further increase realism, our aim here is solely to demonstrate the model’s applicability for inverse parameter retrieval. A comprehensive sensitivity analysis for each input parameter and a full exploration of the nonlinear parameter space lie beyond the scope of this work.

**Table 3:** Optical property parameterizations and reference values for skin and soft tissue.

Parameterization	Skin	Soft tissue
$\mu _s'(\lambda ) = a\,(\lambda /\lambda ^*)^{-b}$	$a = 4.6~\mathrm {mm^{-1}},\; b = 1.4$	$a = 1.9~\mathrm {mm^{-1}},\; b = 1.3$
$\mu _a(\lambda ) = v_{f,\text {blood}} \big [ sO_2 \,\mu _{a,\text {HbO}_2}(\lambda ) + (1 - sO_2)\,\mu _{a,\text {Hb}}(\lambda ) \big ]$	$v_{f,\text {blood}} = 0.01,\; sO_2 = 0.7$	$v_{f,\text {blood}} = 0.05,\; sO_2 = 0.7$
$p_{sb}(\lambda ) = 10^{-5}\!\left[ p_1(\lambda /\lambda ^*) + p_2(\lambda /\lambda ^*)^2 + p_3(\lambda /\lambda ^*)^3\right]$	TTHG: $g_f = 0.8 \rightarrow 0.7,\; g_b = -0.05,\; \alpha = 0.8$	

## Results

### Model parameters and forward errors

After fitting the model separately to the SFR and iSFR datasets for each NA, the optimal parameter values and their 95% confidence intervals were obtained, as summarized in Table 4. The table also lists the original SFR parameters reported by Post *et al.*, for which $b_1$, $b_2$, and the overall median absolute error were reported to be NA-independent. Minor deviations between our fitted parameters and those of Post *et al.* can be attributed to differences in the explored parameter ranges, MC implementations (see Supplementary Materials), and fitting methodology.

**Table 4:** Fitted model parameters for both SFR and iSFR configurations across NAs. Results from Post *et al.* are shown for comparison, with their $b_1$, $b_2$, and overall median absolute error reported as NA-independent. For each parameter, the 95% confidence interval obtained after fitting is indicated by the ± values. The reported 95% confidence intervals are approximate local intervals derived from the Jacobian of the nonlinear least-squares fit. The column labeled “Error (%)” represents the NA-specific median absolute error across the entire parameter space (Table 1 and Table 2) for the corresponding row. For iSFR, these values happen to round to 6.2% for all reported NAs.

	NA	$\boldsymbol{a_1}$	$\boldsymbol{a_2}$	$\boldsymbol{a_3}$	$\boldsymbol{b_1}$	$\boldsymbol{b_2}$	Error (%)
*SFR – Post et al.*							
SFR (Post)	0.10	1.130 $(\pm 0.006)$	4427 $(\pm 87)$	0.785 $(\pm 0.003)$	1.17 $(\pm 0.004)$	0.57 $(\pm 0.001)$	
SFR (Post)	0.22	1.119 $(\pm 0.005)$	3065 $(\pm 50)$	0.750 $(\pm 0.003)$	1.17 $(\pm 0.004)$	0.57 $(\pm 0.001)$	5.6
SFR (Post)	0.50	1.098 $(\pm 0.005)$	1461 $(\pm 22)$	0.684 $(\pm 0.002)$	1.17 $(\pm 0.004)$	0.57 $(\pm 0.001)$	
*SFR – this work*							
SFR (ours)	0.10	1.241 $(\pm 0.002)$	4649 $(\pm 29)$	0.785 $(\pm 0.001)$	1.154 $(\pm 0.001)$	0.565 $(\pm 0.001)$	3.9
SFR (ours)	0.22	1.226 $(\pm 0.001)$	3299 $(\pm 19)$	0.751 $(\pm 0.001)$	1.171 $(\pm 0.001)$	0.564 $(\pm 0.001)$	4.1
SFR (ours)	0.39	1.206 $(\pm 0.002)$	2090 $(\pm 13)$	0.708 $(\pm 0.001)$	1.200 $(\pm 0.001)$	0.562 $(\pm 0.001)$	4.3
SFR (ours)	0.50	1.197 $(\pm 0.002)$	1610 $(\pm 10)$	0.686 $(\pm 0.001)$	1.222 $(\pm 0.001)$	0.560 $(\pm 0.001)$	4.4
*iSFR – this work*							
iSFR (ours)	0.01	0.986 $(\pm 0.001)$	6030 $(\pm 90)$	0.874 $(\pm 0.002)$	1.139 $(\pm 0.002)$	0.567 $(\pm 0.001)$	6.2
iSFR (ours)	0.025	0.987 $(\pm 0.001)$	5801 $(\pm 86)$	0.871 $(\pm 0.002)$	1.146 $(\pm 0.002)$	0.566 $(\pm 0.001)$	6.2
iSFR (ours)	0.05	0.988 $(\pm 0.001)$	5591 $(\pm 83)$	0.867 $(\pm 0.002)$	1.150 $(\pm 0.002)$	0.566 $(\pm 0.001)$	6.2
iSFR (ours)	0.10	0.988 $(\pm 0.001)$	5178 $(\pm 77)$	0.860 $(\pm 0.002)$	1.158 $(\pm 0.002)$	0.566 $(\pm 0.001)$	6.2
iSFR (ours)	0.22	0.987 $(\pm 0.001)$	4125 $(\pm 62)$	0.839 $(\pm 0.002)$	1.175 $(\pm 0.002)$	0.564 $(\pm 0.001)$	6.2

**Fig. 2:**
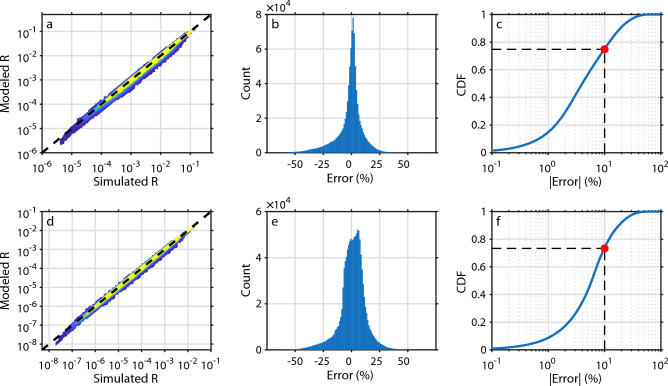
Model performance for SFR (**a–c**) and iSFR (**d–f**). (**a,d**) Log–log scatter plots of model-predicted reflectance versus Monte Carlo simulations across all NAs. Point density is color-coded on a logarithmic scale to indicate regions of high data concentration. (**b,e**) Distribution of relative errors. (**c,f**) Cumulative distribution functions (CDFs) of absolute error. Red markers indicate that 73% (iSFR) and 75% (SFR) of all predictions deviate by less than 10% from the Monte Carlo reference.

To evaluate model accuracy, we compared predicted reflectance values with MC–simulated ground truth across the full parameter space. These error metrics were evaluated on the same MC-simulated datasets used for parameter fitting and therefore quantify within-dataset residual model discrepancy rather than performance on an independent validation set. Because the parameter space was sampled densely and the model contains only a small number of fitted parameters, substantial overfitting is unlikely, although this was not tested explicitly. For SFR, the median absolute error was 4.2%, closely matching the 5.6% error reported by Post *et al.* For iSFR, we observed a modest increase in the median absolute error to 6.2%. Figure 2 shows predicted versus simulated reflectance values for both modalities. The left panels (**a,d**) present log–log scatter plots of model predictions against Monte Carlo simulations. The middle panels (**b,e**) display the corresponding error distributions, while the bottom panels (**c,f**) show cumulative distribution functions (CDFs) of the absolute error. For SFR, 75% of the dataset falls within 10% error, compared with 73% for iSFR. These results confirm that Eqs. (6)–(12) accurately capture the reflectance scaling behavior across a broad range of optical and geometrical conditions, validating the model’s applicability to both contact (SFR) and non-contact (iSFR) geometries.

### Inverse parameter retrieval

**Fig. 3:**
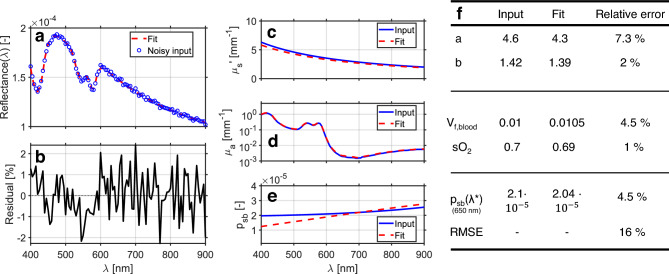
Example inverse-model fit for a single simulated reflectance spectrum ($500~\mu \textrm{m}$ spot size, $\textrm{NA} = 0.05$) representative of skin optical properties. (**a**) Noisy input spectrum with 1% RMS Gaussian noise (blue) and best-fit model prediction (red dashed). (**b**) Residuals between the noisy spectrum and the fitted model. (**c**) Retrieved versus true reduced scattering coefficient $\mu _s'(\lambda )$. (**d**) Retrieved versus true absorption coefficient $\mu _a(\lambda )$. (**e**) Retrieved versus true phase-function descriptor $p_{sb}(\lambda )$. (**f**) Retrieved versus true parameter values for $a$, $b$, $v_{f,\text {blood}}$, and $sO_2$, together with their relative errors. The descriptor $p_{sb}(\lambda )$ is represented by a polynomial; therefore, fit quality is summarized using the RMSE of $p_{sb}(\lambda )$ over the fitted wavelength range and the resulting fit result evaluated at $\lambda ^*$.

Figure 3 illustrates the outcome of a *single* inverse optimization, serving to visualize how the model fits an individual noisy reflectance spectrum and to provide intuition for the magnitude and spectral structure of typical retrieval errors at a realistic noise level. Gaussian noise with an RMS amplitude of 1% was added to the simulated spectrum, after which the inverse model was used to retrieve the wavelength-dependent optical properties $\mu _s'(\lambda )$, $\mu _a(\lambda )$, and $p_{sb}(\lambda )$. Figure 3(**a**) shows the noisy input spectrum together with the best-fit model prediction, while Fig. 3(**b**) displays the corresponding residuals. Panels (**c**)–(**e**) compare the retrieved wavelength-dependent optical properties with the ground-truth inputs used in the simulation. The resulting parameter estimates are summarized quantitatively in Fig. 3(**f**). In this example, all four parameters are recovered with relative errors around 10%. The phase-function descriptor $p_{sb}(\lambda )$ is represented using a polynomial model; consequently, fit quality is more meaningfully quantified by the root-mean-square error (RMSE) of $p_{sb}(\lambda )$ over the fitted wavelength range and by the absolute error at the reference wavelength $\lambda ^*$, rather than by individual polynomial coefficients.

**Fig. 4:**
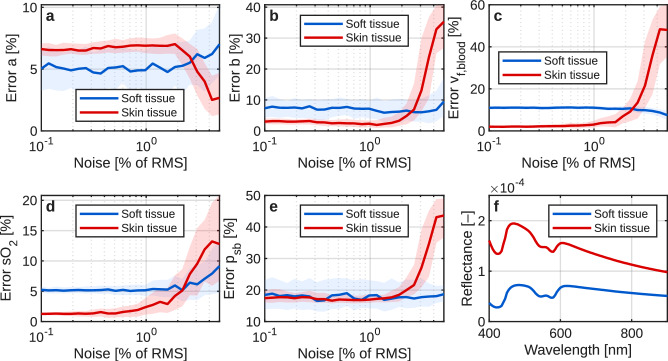
Inverse-model performance as a function of noise level for two tissue types: soft tissue (blue) and skin (red). Each parameter set was fitted 250 times per noise level. Solid lines indicate median relative errors, while shaded regions represent the interquartile range. (**a,b**) Relative errors for the scattering parameters $a$ and $b$. (**c,d**) Relative errors for the absorption parameters $v_{f,\text {blood}}$ and oxygen saturation $sO_2$. (**e**) Relative RMSE for the phase-function descriptor $p_{sb}$. (**f**) Noise-free reference reflectance spectra. Reliable inverse retrieval is maintained up to approximately 3% RMS noise. Persistent low-noise error offsets reflect systematic model inaccuracies, while increased spread and apparent error reductions at higher noise levels result from parameter compensation and convergence to local minima.

While Fig. 3 illustrates the behavior of a single optimization, Fig. 4 summarizes the inverse performance across all simulated noise levels and both tissue types. For each noise level, 250 independent noisy spectra were generated and fitted, yielding distributions of relative parameter errors rather than single values. Fig. 4 shows the median relative error for each retrieved parameter as a function of noise level, together with the interquartile range, which reflects variability arising from noise realizations and local optimization behavior. Panels (**a,b**) report the relative errors for the scattering parameters $a$ and $b$, panels (**c,d**) for the absorption parameters $v_{f,\text {blood}}$ and $sO_2$, and panel (**e**) for the phase-function descriptor $p_{sb}$, expressed as the relative RMSE over wavelength. Panel (**f**) shows the corresponding noise-free reference reflectance spectra for both tissue types. For realistic noise levels of approximately 0.5–1% RMS, most parameter-estimation errors remain below 10%, indicating proof-of-concept inverse performance. Notably, the retrieval errors do not converge to zero in the low-noise limit but instead exhibit a stable offset. This residual error reflects systematic model inaccuracies, as characterized in Fig. 2, which propagate into a bias in the retrieved parameters even in the absence of measurement noise. At higher noise levels (above $\sim$3% RMS), parameter estimates begin to diverge and variability increases substantially. Apparent reductions in the median error for certain parameters at high noise levels (e.g., $a$ for skin and $v_{f,\text {blood}}$ for soft tissue) arise from parameter compensation and convergence to local minima, rather than from improved parameter identifiability.

While these results demonstrate robust inverse performance under realistic noise conditions, a comprehensive evaluation across the full parameter space, including additional tissue types, geometries, and more extreme noise regimes, lies beyond the scope of this work. Future studies will include a detailed sensitivity and identifiability analysis to quantify how model inaccuracies and measurement noise propagate into uncertainties in retrieved optical and physiological parameters.

## Discussion

This work extends the established single-fiber reflectance (SFR) modeling framework to imaging SFR (iSFR), enabling quantitative extraction of optical properties in a non-contact, optically imaged geometry. Using large-scale Monte Carlo simulations combined with an efficient distance-based reflectance computation, we derived and fitted (i)SFR-specific model parameters across a broad range of optical properties and system geometries. The resulting forward model achieves a median absolute error of 6.2% for iSFR and 4.2% for SFR, and inverse fitting demonstrates recovery of absorption and scattering parameterizations to within approximately 10% under realistic noise levels. Together, these results show that the SFR modeling framework remains applicable in non-contact configurations after re-optimization of the empirical parameters, providing a quantitative foundation for iSFR and enabling spatially resolved mapping of optical properties beyond the point-based constraints of contact SFR. Nevertheless, several theoretical and experimental challenges remain and are discussed in the remainder of this section.

### Model errors and scaling relationships

The proposed model accurately captures the dominant scaling behavior of reflectance with respect to optical and geometrical parameters over the majority of the investigated parameter space. As shown in Fig. 2(c–f), a small subset of parameter combinations nevertheless exhibits noticeably larger residual errors. These deviations occur primarily in edge cases corresponding to more extreme parameter combinations. Closer inspection reveals two secondary effects that contribute to these increased errors and are not explicitly captured in the current model formulation.

First, the exponential absorption correction term in Eq. 7 is modeled as a function of the ratio $\mu _a / \mu _s'$. While this approximation performs well in most cases, we find that reflectance is more accurately described by a two-variable dependence of the form $f\!\left( \tfrac{\mu _a}{\mu _s'}, \tfrac{p_{sb}}{(\mu _s' \cdot d_f)^2}\right)$ (see Supplementary Section 4). Second, the ratio between subdiffuse and diffuse reflectance contributions, modeled in Eq. 7 as a power law in $p_{sb} / (\mu _s' \cdot d_f)^2$, implicitly assumes that in the limit $\mu _s' \cdot d_f \rightarrow \infty$ all detected photons become diffuse and $R_{\text {total}} \rightarrow R_{\text {diffuse}}$. While this assumption is generally valid, our simulations indicate that even in this highly diffuse regime a small residual subdiffuse contribution remains. As a result, the ratio $X$ does not strictly reach zero, and the reflectance retains a weak dependence on the detailed phase-function shape that is not fully captured by diffusion theory (see Supplementary Section 4). In principle, this behavior could be addressed by extending the model, for example, by allowing the power-law description of $X$ to saturate at small values of $p_{sb} / (\mu _s' \cdot d_f)$. Exploratory tests of more complex formulations that incorporate these secondary dependencies yielded only minor reductions in model error while increasing parameterization complexity and inversion cost. Given that the associated errors occur primarily in edge cases and constitute only a small fraction of the dataset, we retain the simpler formulation of Post *et al.*^23,24^ to preserve computational efficiency and interpretability. Consequently, these secondary effects were not explicitly incorporated into the final model formulation and do not affect the main conclusions. Nevertheless, they provide additional physical insight into light–tissue interactions in the subdiffuse regime.

A further trend was observed between the fitted parameter $a_2$ and the NA, which appeared approximately linear over most NAs. This suggests that NA could, in principle, be incorporated directly into the model through a dependence of $a_2$ on NA. Implementing this approach, however, led to a slight increase in overall model error, likely because our NA sampling is sparse. Nevertheless, the empirical trend remains useful in practice: interpolating $a_2$ as a function of NA may enable reflectance prediction for iSFR systems with intermediate NAs that were not explicitly simulated. The relationship between $a_2$ and NA is further described in Supplementary Section 3.

### Parameter retrieval

The inverse retrieval results for simulated reflectance spectra from two representative tissue types demonstrate the model’s ability to recover optical parameters and confirm its correct inverse behavior. However, care should be taken when translating these results to real-world performance, as they were obtained under specific modeling assumptions and represent only a subset of the explored parameter and geometry space. In this proof-of-concept demonstration, only two chromophores (oxygenated and deoxygenated hemoglobin) were included. In practice, modeling different tissue types may require the inclusion of additional chromophores such as melanin, water, and lipids to provide realistic coverage across the visible and near-infrared spectrum. Including multiple chromophores poses no conceptual limitation, as their absorption spectra can be linearly combined and weighted by concentration to match the retrieved $\mu _a(\lambda )$. However, increasing the number of fitted chromophores may introduce additional uncertainty. We also assumed a single homogeneous tissue layer with fixed refractive indices. While this is a simplification, it is partially justified by the shallow probing depth of (i)SFR, which typically spans only a few hundred micrometers. Within this limited sampling volume, assuming homogeneity is a reasonable approximation. Nevertheless, when local heterogeneity becomes significant, this approximation can lead to increased errors and uncertainties in retrieved optical properties. In principle, the presented MC framework can be extended to simulate radially symmetric multilayered media. The efficiency gained through the SIA would help offset the additional computational complexity required to simulate such layered structures. At the same time, introducing multilayered tissue models and corresponding inverse problems increases model complexity and parameter uncertainty. Future work should therefore assess whether such extensions provide a meaningful benefit over the homogeneous tissue assumption for (i)SFR.

All inverse tests were performed for a single geometry ($500~\mu \textrm{m}$ spot size, $\textrm{NA} = 0.05$). Further work is required to evaluate model behavior across geometries, as both fiber diameter and NA influence probing depth and forward-model accuracy, thereby affecting retrieval reliability. Such analyses will also clarify under which conditions multi-diameter acquisition (MD-(i)SFR) becomes advantageous. In conventional quantitative SFR, the use of multiple fiber diameters improves parameter separability^14^. In the present study, we observe that for the investigated geometries, optical properties, and noise levels, single-fiber inverse retrieval using a multistart optimization approach already achieves error levels comparable to those reported for multi-diameter SFR^24^. To better understand the theoretical performance limits of the model, future work will include a biased Cramér–Rao lower bound analysis. This will quantify the achievable precision, accuracy, and sensitivity of the retrieved parameters under realistic noise and model assumptions, including measurement uncertainties such as geometry mismatch arising from sample-height variations, which are essential steps toward establishing (i)SFR as a reliable quantitative technique in both biomedical and materials-science applications.

### Phase-functions and $p_{sb}$

The phase-function descriptor $p_{sb}$ provides a pragmatic means of incorporating phase-function effects into subdiffuse reflectance modeling. Nevertheless, a comprehensive and physically grounded treatment of phase functions in the subdiffuse regime remains lacking, including forward modeling, inverse parameterization, and biological characterization. The current definition of $p_{sb}$, based on empirically optimized angular integration limits, reflects this incomplete understanding and consequently limits physical interpretability. The use of fixed angular bounds may neglect information contained in other regions of the scattering phase function, as broader angular ranges could encode additional features relevant to subdiffuse photon transport. Although a power-law relationship of the form $X \sim p_{sb}^{0.75}$ is consistently observed, its fundamental physical origin remains unclear. A wavelength-dependent formulation of $p_{sb}$ poses an additional challenge. Experimental data on the spectral variation of biological phase functions are scarce^46^, limiting efforts to constrain and validate spectrally resolved phase-function descriptors. In this context, it is notable that diagnostic contrast has previously been demonstrated using a single, wavelength-independent value of $p_{sb}$ evaluated at a fixed wavelength^14^. This suggests that, for certain diagnostic tasks, treating $p_{sb}$ as spectrally invariant may already be sufficient, while substantially reducing the number of fit parameters and mitigating parameter competition with $\mu _s'(\lambda )$. Overall, the limited understanding of how biological phase functions vary across tissue types, pathological states, and wavelengths highlights a broader gap in subdiffuse light–tissue modeling. Addressing this gap will require advances in experimental phase-function characterization alongside the development of more physically motivated phase-function descriptors and inversion strategies. Such progress represents a key opportunity for subdiffuse techniques, which are inherently sensitive to phase-function structure that may contain diagnostically relevant information beyond conventional scattering parameters.

### Experimental challenges

Several technical challenges must be addressed before quantitative parameter extraction using iSFR can be experimentally validated. A dedicated validation setup is currently being designed, but reliable parameter retrieval ultimately depends on the quality and stability of the acquired spectra. Key requirements include the development of a robust calibration protocol (established for SFR^53^ but not directly applicable to iSFR), minimizing or explicitly modeling specular reflections, quantifying measurement uncertainty, ensuring optical power stability^56^, compensating for sample-height variations (which can induce geometry mismatch by altering the effective tissue-plane NA and projected spot size), and improving the speed and robustness of the inverse-fitting algorithm.

Moreover, for clinical implementation, measurement speed is a critical constraint, which limits the use of long spectrometer integration times. At the same time, optical power is restricted by safety considerations, precluding straightforward compensation through increased illumination intensity. As a result, the achievable signal-to-noise ratio is largely governed by the illumination and collection geometry. In practice, both the NA and the spot size can be controlled through the optical design and directly determine the number of detected photons, requiring a careful balance between sensitivity, spatial resolution, and acquisition speed. Extremely low NA values and small spot sizes, while attractive for multimodal OCT integration and high spatial resolution, may yield reflectance levels that are insufficiently above the noise floor for realistic power levels and acquisition times. The presented model, together with a future sensitivity analysis, will enable a systematic assessment of these fundamental trade-offs, including the potential benefits and limitations of multispectral acquisition strategies. Addressing these aspects will form the focus of future work and is essential for meaningful model validation and for assessing the practical performance of iSFR under realistic experimental and clinical conditions.

## Conclusion

We present a quantitative model for imaging SFR that extends and validates the established SFR framework for non-contact configurations. By benchmarking against the model of Post *et al.*^23,24^, we demonstrate that the same underlying formulation remains valid for iSFR, provided that the empirical model parameters are re-optimized to account for boundary effects and geometric differences. Using an efficient distance-based reflectance computation, we generated a large dataset of Monte Carlo simulations spanning realistic optical and geometrical parameter ranges. Fitting this dataset yielded model parameters with median absolute errors of 4% for SFR and 6% for iSFR. Two simulation-based inverse-retrieval demonstrations further showed that the model can recover wavelength-dependent absorption and scattering properties from noisy simulated spectra, providing a proof-of-concept for quantitative inverse modeling. Nevertheless, open questions remain regarding theoretical scaling limits, parameter sensitivity, and experimental implementation. Overall, this work establishes a quantitative and computationally efficient foundation for iSFR spectroscopy, enabling high-resolution, spatially resolved mapping of tissue optical properties in a single-fiber, non-contact configuration.

## Supplementary Information

Supplementary Information.

## Data Availability

Data generated during this study are not publicly available due to their size but are available from the corresponding author upon reasonable request. Example code demonstrating implementation details is available on GitHub: https://github.com/robinvanzutphen/iSFR-example.

## References

[1] Kortüm, G. *Reflectance Spectroscopy: Principles, Methods, Applications* (Springer, 1969).

[2] Evers, D., Hendriks, B., Lucassen, G. & Ruers, T. Optical spectroscopy: Current advances and future applications in cancer diagnostics and therapy. *Future Oncol.* **8**, 307–320 (2012). 22409466 10.2217/fon.12.15

[3] Akter, S., Hossain, M. G., Nishidate, I., Hazama, H. & Awazu, K. Medical applications of reflectance spectroscopy in the diffusive and sub-diffusive regimes. *J. Near Infrared Spectrosc.* **26**, 337–350 (2018).

[4] Lu, B., Dao, P. D., Liu, J., He, Y. & Shang, J. Recent advances of hyperspectral imaging technology and applications in agriculture. *Remote Sensing* **12**, 2659 (2020).

[5] Nduwamungu, C., Ziadi, N., Parent, L. -É., Tremblay, G. F. & Thuriès, L. Opportunities for, and limitations of, near infrared reflectance spectroscopy applications in soil analysis: A review. *Can. J. Soil Sci.* **89**, 531–541 (2009).

[6] Edelman, G. J., Gaston, E., van Leeuwen, T. G., Cullen, P. & Aalders, M. C. Hyperspectral imaging for non-contact analysis of forensic traces. *Forensic Sci. Int.* **223**, 28–39 (2012). 23088824 10.1016/j.forsciint.2012.09.012

[7] Zhang, X. U., Faber, D. J., Post, A. L., van Leeuwen, T. G. & Sterenborg, H. J. Refractive index measurement using single fiber reflectance spectroscopy. *J. Biophotonics* **12**, e201900019 (2019). 30908898 10.1002/jbio.201900019 PMC7065624

[8] Hariri Tabrizi, S., Mahmoud Reza Aghamiri, S., Farzaneh, F., Amelink, A. & Sterenborg, H. J. Single fiber reflectance spectroscopy on cervical premalignancies: the potential for reduction of the number of unnecessary biopsies. *J. Biomed. Opt.* **18**, 017002–017002 (2013). 10.1117/1.JBO.18.1.017002 23292613

[9] Kanick, S. C. et al. Integration of single-fiber reflectance spectroscopy into ultrasound-guided endoscopic lung cancer staging of mediastinal lymph nodes. *J. Biomed. Opt.* **15**, 017004–017004 (2010). 20210478 10.1117/1.3290822

[10] Fawzy, Y. S., Petek, M., Tercelj, M. & Zeng, H. In vivo assessment and evaluation of lung tissue morphologic and physiological changes from non-contact endoscopic reflectance spectroscopy for improving lung cancer detection. *J. Biomed. Opt.* **11**, 044003–044003 (2006). 16965160 10.1117/1.2337529

[11] van Manen, L. et al. Single fiber reflectance spectroscopy for pancreatic cancer detection during endoscopic ultrasound guided fine needle biopsy: a prospective cohort study. *Int. J. Med. Sci.* **19**, 205 (2022). 35165506 10.7150/ijms.65364 PMC8795801

[12] Yu, L., Wu, Y., Dunn, J. F. & Murari, K. In-vivo monitoring of tissue oxygen saturation in deep brain structures using a single fiber optical system. *Biomed. Opt. Express* **7**, 4685–4694 (2016). 27896007 10.1364/BOE.7.004685 PMC5119607

[13] Bugter, O., Hardillo, J. A., Baatenburg de Jong, R. J., Amelink, A. & Robinson, D. J. Optical pre-screening for laryngeal cancer using reflectance spectroscopy of the buccal mucosa. *Biomed. Opt. Express* **9**, 4665–4678 (2018). 30319894 10.1364/BOE.9.004665 PMC6179391

[14] Post, A. L. et al. Toward improved endoscopic surveillance with multidiameter single fiber reflectance spectroscopy in patients with barrett’s esophagus. *J. Biophotonics* **14**, e202000351 (2021). 33410602 10.1002/jbio.202000351

[15] Zhang, X. U., Faber, D. J., van Leeuwen, T. G. & Sterenborg, H. J. Effect of probe pressure on skin tissue optical properties measurement using multi-diameter single fiber reflectance spectroscopy. *J. Phys. Photon.* **2**, 034008 (2020).

[16] Tabrizi, S. H. & Shakibaei, A. A. The effect of probe pressure on in vivo single fiber reflectance spectroscopy. *J. Lasers Med. Sci.* **7**, 233 (2016). 28491258 10.15171/jlms.2016.41 PMC5415500

[17] Attendu, X. et al. Combined optical coherence tomography and broadband single fiber reflectance spectroscopy. *Multimodal Biomed. Imaging XVI* **11634**, 1163403 (2021).

[18] Attendu, X. et al. All-reflective tethered capsule endoscope for multimodal optical coherence tomography in the esophagus. *J. Biomed. Opt.* **29**, 096003–096003 (2024). 39301278 10.1117/1.JBO.29.9.096003 PMC11412323

[19] Attendu, X., Bourget, M.-H., de Sivry-Houle, M. P. & Boudoux, C. Coregistered optical coherence tomography and frequency-encoded multispectral imaging for spectrally sparse color imaging. *J. Biomed. Opt.* **25**, 032008–032008 (2020). 10.1117/1.JBO.25.3.032008 PMC7011031 31755250

[20] Guay-Lord, R. et al. Combined optical coherence tomography and hyperspectral imaging using a double-clad fiber coupler. *J. Biomed. Opt.* **21**, 116008–116008 (2016). 27829103 10.1117/1.JBO.21.11.116008

[21] Wartak, A. et al. Multimodality optical coherence tomography based tethered capsule endomicroscopy for upper gastrointestinal tract imaging. In *Optical Coherence Tomography and Coherence Domain Optical Methods in Biomedicine XXV* Vol. 11630 (ed. Wartak, A.) 116300 (SPIE, 2021).

[22] Buenconsejo, A. L. et al. Submillimeter diameter rotary-pullback fiber-optic endoscope for narrowband red-green-blue reflectance, optical coherence tomography, and autofluorescence in vivo imaging. *J. Biomed. Opt.* **25**, 032005–032005 (2020). 10.1117/1.JBO.25.3.032005 PMC7010984 31650742

[23] Post, A. L., Sterenborg, H. J., Woltjer, F. G., van Leeuwen, T. G. & Faber, D. J. Subdiffuse scattering model for single fiber reflectance spectroscopy. *J. Biomed. Opt.* **25**, 015001–015001 (2020). 31920047 10.1117/1.JBO.25.1.015001 PMC7008500

[24] Post, A. L., Faber, D. J., Sterenborg, H. J. & van Leeuwen, T. G. Subdiffuse scattering and absorption model for single fiber reflectance spectroscopy. *Biomed. Opt. Express* **11**, 6620–6633 (2020). 33282512 10.1364/BOE.402466 PMC7687961

[25] Yoon, G., Prahl, S. A. & Welch, A. J. Accuracies of the diffusion approximation and its similarity relations for laser irradiated biological media. *Appl. Opt.* **28**, 2250–2255 (1989). 20555507 10.1364/AO.28.002250

[26] Martelli, F., Bassani, M., Alianelli, L., Zangheri, L. & Zaccanti, G. Accuracy of the diffusion equation to describe photon migration through an infinite medium: numerical and experimental investigation. *Phys. Med. Biol.* **45**, 1359–1373 (2000). 10843109 10.1088/0031-9155/45/5/318

[27] Kienle, A., Forster, F. K. & Hibst, R. Influence of the phase function on determination of the optical properties of biological tissue by spatially resolved reflectance. *Opt. Lett.* **26**, 1571–1573 (2001). 18049666 10.1364/ol.26.001571

[28] Bodenschatz, N., Krauter, P., Liemert, A. & Kienle, A. Quantifying phase function influence in subdiffusively backscattered light. *J. Biomed. Opt.* **21**, 035002–035002 (2016). 10.1117/1.JBO.21.3.035002 26968384

[29] Mourant, J. R., Boyer, J., Hielscher, A. H. & Bigio, I. J. Influence of the scattering phase function on light transport measurements in turbid media performed with small source-detector separations. *Opt. Lett.* **21**, 546–548 (1996). 19865467 10.1364/ol.21.000546

[30] Vitkin, E. et al. Photon diffusion near the point-of-entry in anisotropically scattering turbid media. *Nat. Commun.* **2**, 587 (2011). 22158442 10.1038/ncomms1599 PMC3370954

[31] Jia, M. et al. Virtual-source diffusion approximation for enhanced near-field modeling of photon-migration in low-albedo medium. *Opt. Express* **23**, 1337–1352 (2015). 25835892 10.1364/OE.23.001337

[32] Piao, D. & Patel, S. Simple empirical master-slave dual-source configuration within the diffusion approximation enhances modeling of spatially resolved diffuse reflectance at short-path and with low scattering from a semi-infinite homogeneous medium. *Appl. Opt.* **56**, 1447–1452 (2017). 10.1364/AO.57.007942 30462064

[33] Sun, T. & Piao, D. Diffuse photon remission associated with the center-illuminated-area-detection geometry: Part i, an approach to the steady-state model. *Appl. Opt.* **61**, 9143–9153 (2022). 36607047 10.1364/AO.468342

[34] Venugopalan, V., You, J. & Tromberg, B. Radiative transport in the diffusion approximation: an extension for highly absorbing media and small source-detector separations. *Phys. Rev. E* **58**, 2395 (1998).

[35] Hull, E. L. & Foster, T. H. Steady-state reflectance spectroscopy in the p3 approximation. *J. Opt. Soc. Am. A* **18**, 584–599 (2001).

[36] Wilk, L. S. & Aalders, M. C. Bridging photon transport regimes in turbid media by accounting for the finite speed of light. *Phys. Rev. Appl.* **23**, 054050 (2025).

[37] Kanick, S., Robinson, D., Sterenborg, H. & Amelink, A. Monte carlo analysis of single fiber reflectance spectroscopy: photon path length and sampling depth. *Phys. Med. Biol.* **54**, 6991–7008 (2009). 19887712 10.1088/0031-9155/54/22/016

[38] Kanick, S. C., Gamm, U. A., Sterenborg, H. J., Robinson, D. J. & Amelink, A. Method to quantitatively estimate wavelength-dependent scattering properties from multidiameter single fiber reflectance spectra measured in a turbid medium. *Opt. Lett.* **36**, 2997–2999 (2011). 21808384 10.1364/OL.36.002997

[39] Kanick, S. C., Robinson, D. J., Sterenborg, H. J. & Amelink, A. Method to quantitate absorption coefficients from single fiber reflectance spectra without knowledge of the scattering properties. *Opt. Lett.* **36**, 2791–2793 (2011). 21808314 10.1364/OL.36.002791

[40] Bevilacqua, F. & Depeursinge, C. Monte carlo study of diffuse reflectance at source-detector separations close to one transport mean free path. *J. Opt. Soc. Am. A* **16**, 2935–2945 (1999).

[41] Naglič, P., Pernuš, F., Likar, B. & Bürmen, M. Adopting higher-order similarity relations for improved estimation of optical properties from subdiffusive reflectance. *Opt. Lett.* **42**, 1357–1360 (2017). 28362768 10.1364/OL.42.001357

[42] Faber, D. J., Post, A. L., Sterenborg, H. J. & van Leeuwen, T. G. Analytical model for diffuse reflectance in single fiber reflectance spectroscopy. *Opt. Lett.* **45**, 2078–2081 (2020). 32236072 10.1364/OL.385845

[43] Farrell, T. J., Patterson, M. S. & Wilson, B. A diffusion theory model of spatially resolved, steady-state diffuse reflectance for the noninvasive determination of tissue optical properties in vivo. *Med. Phys.* **19**, 879–888 (1992). 1518476 10.1118/1.596777

[44] Martelli, F., Binzoni, T., Del Bianco, S., Liemert, A. & Kienle, A. *Light Propagation Through Biological Tissue and Other Diffusive Media: Theory, Solutions, and Validations* (SPIE Bellingham, 2022).

[45] Jacques, S. L. Optical properties of biological tissues: A review. *Phys. Med. Biol.* **58**, R37–R61 (2013). 23666068 10.1088/0031-9155/58/11/R37

[46] Witteveen, M. et al. Opportunities and pitfalls in (sub) diffuse reflectance spectroscopy. *Front. Photon.* **3**, 964719 (2022).

[47] Jacques, S. L., Alter, C. & Prahl, S. A. Angular dependence of hene laser light scattering by human dermis. *Lasers Life Sci.* **1**, 309–333 (1987).

[48] Pfeiffer, N. & Chapman, G. H. Successive order, multiple scattering of two-term henyey-greenstein phase functions. *Opt. Express* **16**, 13637–13642 (2008). 18772974 10.1364/oe.16.013637

[49] Reynolds, L. & McCormick, N. Approximate two-parameter phase function for light scattering. *J. Opt. Soc. Am.* **70**, 1206–1212 (1980).

[50] Fang, Q. & Boas, D. A. Monte carlo simulation of photon migration in 3d turbid media accelerated by graphics processing units. *Opt. Express* **17**, 20178–20190 (2009). 19997242 10.1364/OE.17.020178 PMC2863034

[51] Wang, L., Jacques, S. L. & Zheng, L. Conv-convolution for responses to a finite diameter photon beam incident on multi-layered tissues. *Comput. Methods Programs Biomed.* **54**, 141–150 (1997). 9421660 10.1016/s0169-2607(97)00021-7

[52] Ahnesjö, A., Saxner, M. & Trepp, A. A pencil beam model for photon dose calculation. *Med. Phys.* **19**, 263–273 (1992). 1584117 10.1118/1.596856

[53] Zhang, X. U., Post, A. L., Faber, D. J., van Leeuwen, T. G. & Sterenborg, H. J. Single fiber reflectance spectroscopy calibration. *J. Biomed. Opt.* **22**, 100502–100502 (2017). 10.1117/1.JBO.22.10.100502 29086543

[54] Bosschaart, N., Edelman, G. J., Aalders, M. C., van Leeuwen, T. G. & Faber, D. J. A literature review and novel theoretical approach on the optical properties of whole blood. *Lasers Med. Sci.* **29**, 453–479 (2014). 24122065 10.1007/s10103-013-1446-7 PMC3953607

[55] Post, A. L., Faber, D. J., Sterenborg, H. J. & van Leeuwen, T. G. Experimental validation of a recently developed model for single-fiber reflectance spectroscopy. *J. Biomed. Opt.* **26**, 025004–025004 (2021). 33641270 10.1117/1.JBO.26.2.025004 PMC7913601

[56] Attendu, X., Benk-Fortin, A., Faber, D. J., Boudoux, C. & van Leeuwen, T. G. Stabilized single-fiber reflectance spectroscopy using wideband multimode circulators. In *Advanced Biomedical and Clinical Diagnostic and Surgical Guidance Systems XXII*, PC128310I (SPIE, 2024).

