# Supplementary material for: Quantitative model for imaging single fiber reflectance spectroscopy
Source: Sci Rep. 2026 Apr 14;16:17300. doi: 10.1038/s41598-026-48855-y (PMC13234268; doi:10.1038/s41598-026-48855-y)
Supplement: Supplementary file 1 — Supplementary Information. [file 41598_2026_48855_MOESM1_ESM.pdf]

Supplementary Materials:
Model Development for Quantitative Imaging Single Fiber Reflectance Spectroscopy

Robin van Zutphen[1,2,*], Ton G. van Leeuwen[1,2,3], and Xavier Attendu[1,2]

[1]Amsterdam UMC location University of Amsterdam, Biomedical Engineering and Physics, Meibergdreef 9, Amsterdam, The Netherlands
[2]Cancer Center Amsterdam, Imaging and Biomarkers, Amsterdam, The Netherlands
[3]Amsterdam Cardiovascular Sciences, Heart failure & arrhythmias, Amsterdam, The Netherlands
[*]r.vanzutphen@amsterdamumc.nl

Contents

1 Validity of the phase-function descriptor p_{sb} for iSFR **2**

2 Reflectance calculations **4**
2.1 Single-Integral Approximation 4
2.2 Algorithm outline 6
2.3 Monte Carlo configuration 9
2.4 Phase function discretization and p_{sb} calculation 10
2.5 Stochastic MC noise 11

3 Results across numerical apertures **12**

4 Additional scaling relationships **13**

5 Inversion routine **15**

1 Validity of the phase-function descriptor p_{sb} for iSFR

The phase-function descriptor p_{sb}, originally introduced for SFR, is defined through forward and backward angular integration limits that were empirically optimized for typical SFR geometries [1]. Imaging SFR (iSFR), however, can be implemented over a substantially wider range of numerical apertures (NAs), raising the question of whether the original integration limits remain optimal and whether p_{sb} remains a suitable descriptor under iSFR conditions. To address this, we followed a methodology analogous to the original derivation of p_{sb} [1].

A wide range of scattering phase functions (Table 2, Main Paper) was simulated in a highly subdiffuse regime with $\mu_a = 0$ and $\mu_s' \cdot d_f = 0.1$, ensuring that the detected reflectance is dominated by semiballistic photons and is therefore maximally sensitive to the phase-function shape. For each phase function, reflectance was computed using Monte Carlo (MC) simulations for both SFR and iSFR geometries. Candidate values for the forward (θ_f) and backward (θ_b) integration limits were systematically varied over the range 1°–90° in steps of 1°. For each (θ_f, θ_b) pair, the suitability of the resulting p_{sb} definition was quantified by evaluating the dispersion of p_{sb} values at fixed reflectance. Reflectance values were grouped into $N_R = 5$ logarithmically spaced reference levels R_k. For each reference level, a reflectance bin was constructed by including all simulations whose reflectance satisfied

$$|\log_{10} R_i - \log_{10} R_k| \leq \log_{10}(1.1), \tag{1}$$

corresponding to a tolerance of ±10% in reflectance. Within each reflectance bin $\mathcal{R}_k$, the dispersion of p_{sb} was defined as the range of p_{sb} values across all phase functions yielding similar reflectance,

$$\Delta p_{sb}(\mathcal{R}_k) = \max_{i \in \mathcal{R}_k} p_{sb,i} - \min_{i \in \mathcal{R}_k} p_{sb,i}. \tag{2}$$

To obtain a relative measure independent of the absolute scale of p_{sb}, this quantity was normalized by the total spread of p_{sb} across all simulated phase functions,

$$\Delta p_{sb}^{\text{tot}} = \max_i p_{sb,i} - \min_i p_{sb,i}. \tag{3}$$

The optimal integration limits were then determined by minimizing the average relative dispersion across all reflectance bins,

$$(\theta_f^*, \theta_b^*) = \arg\min_{\theta_f, \theta_b} \frac{1}{N_R} \sum_{k=1}^{N_R} \frac{\Delta p_{sb}(\mathcal{R}_k; \theta_f, \theta_b)}{\Delta p_{sb}^{\text{tot}}(\theta_f, \theta_b)}. \tag{4}$$

This criterion enforces a near one-to-one relationship between reflectance and p_{sb} in the subdiffuse regime, which is beneficial for stable inverse modeling, as it minimizes phase-function–induced degeneracy at fixed reflectance. A visual explanation of this methodology for determining the optimal integration angles can be found in the original publication [1].

This analysis was repeated for the full set of iSFR numerical apertures considered in this work, {0.01, 0.025, 0.05, 0.10, 0.22}, as well as for conventional SFR NAs for reference. The resulting relative dispersion as a function of the integration angles is shown in Fig. 1. For SFR, the observed trends closely reproduce previously reported results, confirming the consistency of our implementation. The optimal integration limits for each NA are indicated in the figure legends. In all cases, the optimal backward integration angle was found to be $\theta_b = 1°$, while the optimal forward integration angle θ_f exhibited only a weak dependence on NA. For iSFR, a similar trend was observed: as NA decreases, the optimal forward integration angle shifts to smaller values.

In the original SFR formulation, fixed integration limits of $\theta_b = 1°$ and $\theta_f = 23°$ were proposed based on typical clinical SFR fibers with NA ≈ 0.22, and it was shown that the use of fixed limits resulted in only a marginal increase in dispersion compared to NA-specific optimal

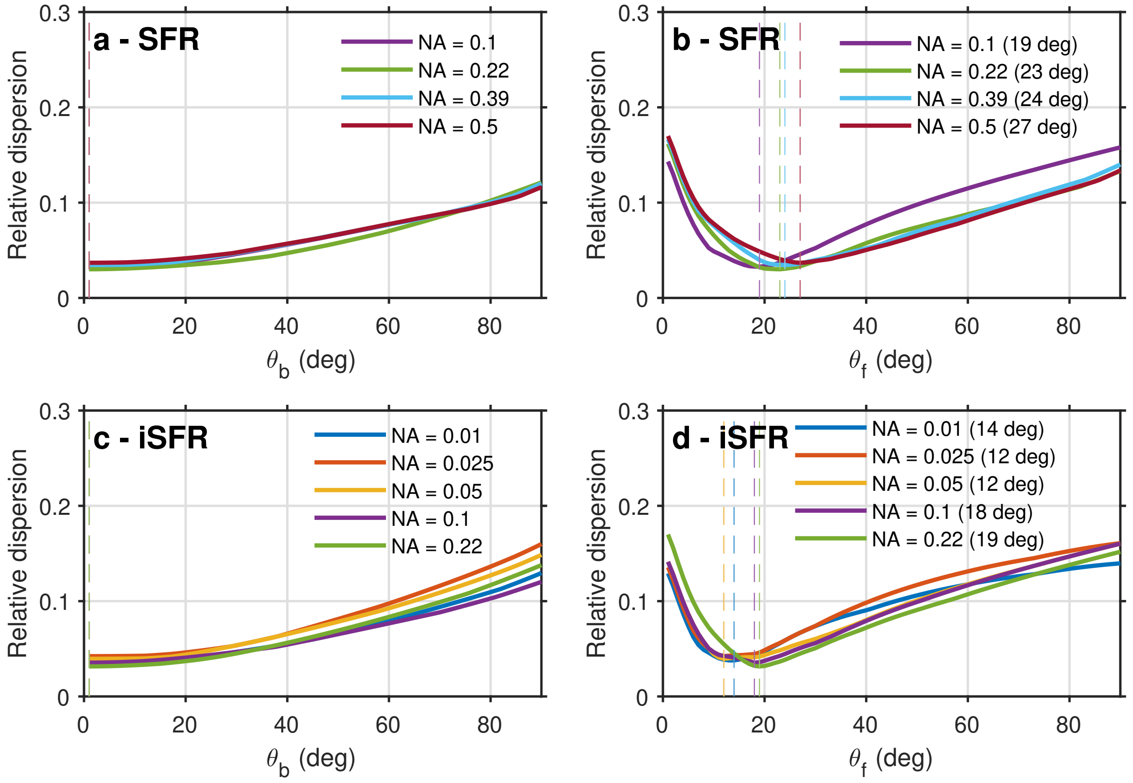

Figure 1: Optimal angular integration limits for minimizing the relative dispersion of p_{sb}. The top row (**a,b**) shows results for SFR, and the bottom row (**c,d**) for iSFR. In both geometries, the optimal backward integration angle (**a,c**) was found to be 1°. While the optimal forward integration angle for iSFR (**d**) was slightly lower than the 23° identified for SFR (**b**), a forward angle of 23° was retained for both modalities to maintain consistency with the original p_{sb} derivation.

values. To evaluate whether NA-dependent integration limits provide a benefit for iSFR, we compared model performance using the NA-specific optimal forward angles (as listed in the figure legend) with performance obtained using the fixed limits $\theta_b = 1°$ and $\theta_f = 23°$. The impact on forward-model accuracy was negligible, with changes in the median forward error remaining below 0.2%. Given this minimal improvement, and to maintain consistency with the original SFR formulation for practical implementation, the fixed integration limits $\theta_b = 1°$ and $\theta_f = 23°$ were retained throughout this work for both SFR and iSFR.

These results demonstrate that, within the subdiffuse regime considered here, the phase-function descriptor p_{sb} provides a near one-to-one mapping between reflectance and phase-function shape for iSFR across the investigated numerical aperture range. As such, p_{sb} remains a suitable parameter for capturing phase-function–induced variations in reflectance under iSFR conditions, without requiring modification of its original definition.

2 Reflectance calculations

2.1 Single-Integral Approximation

To efficiently compute reflectance for finite source–detector geometries, we employ a distance-weighted single-integral approximation (SIA). This approach was originally derived for the *diffuse* reflectance contribution under the diffusion approximation and for identical, uniformly distributed, overlapping source–detector disks [2]. By recognizing that the reflectance depends only on the distance between illumination and detection locations across the fiber face, rather than on their absolute coordinates, the double surface integral arising from finite-area illumination can be reduced to a single integral over the probability distribution of possible source–detector distances. Here, we apply the same geometric reduction to the *total* reflectance obtained from MC simulations, thereby extending the SIA beyond diffusion theory into the subdiffuse regime relevant for (i)SFR.

We restrict the present validation to the configuration used throughout this work: identical, overlapping circular source and detector areas with diameter d_f. For this geometry, the total reflectance can be written as

$$R_{\text{tot}}(d_f) = \frac{\pi}{4}\, d_f^2 \int_0^{d_f} R(\rho)\, p(\rho, d_f)\, \mathrm{d}\rho, \tag{5}$$

where $R(\rho)$ is the total radial reflectance per unit incident power as a function of the radial distance from the source center ρ, obtained from localized quasi–pencil-beam MC simulations (pencil-beam illumination with angular spread defined by the NA), and $p(\rho, d_f)$ is the probability density of distances between two randomly selected points on a disk of diameter d_f. A closed-form expression for $p(\rho, d_f)$ is given by [3]

$$p(\rho, d_f) = \frac{16\rho}{\pi d_f^2} \cos^{-1}\left(\frac{\rho}{d_f}\right) - \frac{16}{\pi d_f^2} \frac{\rho^2}{d_f} \sqrt{1 - \left(\frac{\rho}{d_f}\right)^2}, \qquad 0 \leq \rho \leq d_f. \tag{6}$$

In addition to replacing the analytical diffusion-based reflectance with $R(\rho)$ obtained from MC simulations, we extend the SIA framework to account for finite-NA effects. These effects are incorporated directly by enforcing angular launch and detection constraints within the MC simulations; therefore, no additional corrections are required in the integral formulation itself.

To validate this approximation, we compared reflectance values obtained from explicit MC simulations with finite, overlapping source–detector disks to reflectance computed using the SIA. The comparison was performed for a representative set of optical properties and geometries used throughout the main manuscript, namely $\mu_s' = 5$ mm^{-1}, $\mu_a = 0.1$ mm^{-1}, a Henyey–Greenstein phase function with $g = 0.85$, NA $= 0.1$, and a spot size of 500 μm. In addition, two edge cases were evaluated: one with a very low numerical aperture (NA $= 0.01$) and one with a relatively high-absorption configuration ($\mu_a = 5$ mm^{-1}, $\mu_s' = 0.5$ mm^{-1}).

Figure 2 shows the resulting reflectance values obtained using both approaches. For each of the three validation scenarios, both the explicit full source–detector MC simulation and the corresponding SIA-based reflectance computation were repeated 10^4 times using independent random seeds. Each repeat consisted of a separate MC run with 5×10^7 launched photons, after which the total reflectance was computed for that run. This procedure allowed us to quantify stochastic MC variability and to distinguish it from any systematic difference between the two approaches. In all three scenarios, the relative difference between the mean values is below 0.1%. We attribute this small offset to discretization and numerical integration errors.

This validation demonstrates that, for overlapping source–detector geometries with equal radii, the SIA provides an accurate and computationally efficient means of computing reflectance from a single MC simulation, even when finite-NA effects and subdiffuse photon transport are included. Moreover, because the reflectance is computed solely from photon exit locations and

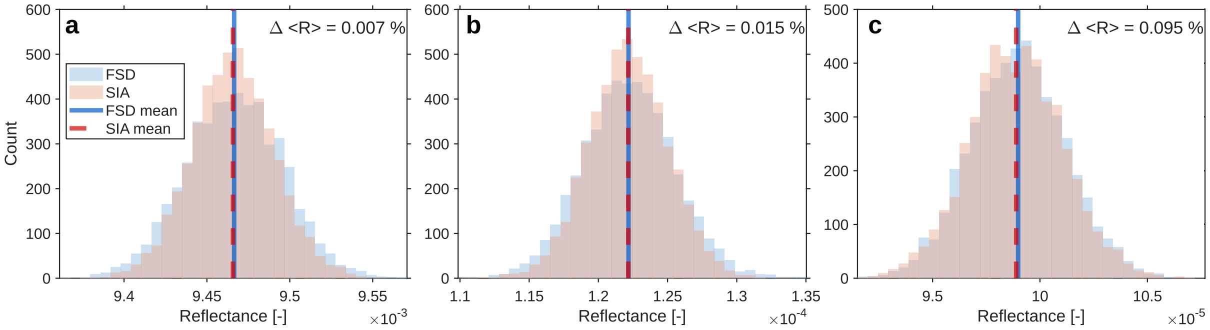

Figure 2: Validation of the single-integral approximation (SIA) against explicit Monte Carlo simulations with finite, overlapping source–detector disks. Shown are the resulting total reflectance distributions obtained using the SIA and full source–detector simulations for **(a)** the representative base case ($\mu_s' = 5$ mm^{-1}, $\mu_a = 0.1$ mm^{-1}, $g = 0.85$, NA $= 0.1$, $d_f = 500$ μm), **(b)** a low-NA edge case (NA $= 0.01$), and **(c)** a high-absorption edge case ($\mu_a = 5$ mm^{-1}, $\mu_s' = 0.5$ mm^{-1}). For each case, both the full source–detector MC simulation and the corresponding SIA result were evaluated in 10^4 independent repeats, each based on a separate MC run with 5×10^7 launched photons. In all scenarios, the mean reflectance values obtained with both methods agree to within 0.1%.

weights, no fluence calculations are required, further improving computational efficiency. Details of the numerical implementation are provided in the following section, along with an example script.

The underlying geometric argument of the SIA is general and can, in principle, be extended to other source–detector configurations, such as concentric disks of unequal radii or non-overlapping geometries, by substituting $p(\rho, d_f)$ with the appropriate distance distribution and adjusting the normalization accordingly. While we verified this behavior in exploratory simulations, these cases are not required for the present work and are therefore not considered further. A systematic treatment of more general source–detector geometries will be presented in future work.

2.2 Algorithm outline

The generation of the reflectance dataset consists of two main stages. In the first stage, detected-photon data are generated according to *Algorithm 1*. For each combination of numerical aperture (NA), reduced scattering coefficient (μ_s'), and phase function $p(\theta)$, the number of detected photons is increased until the coefficient of variation (COV) of the estimated reflectance falls below 1% (see Section 2.5). This requirement is enforced by specifying a minimum number of detected photons, N_{min}, which directly controls the COV of the reflectance estimate. Because the smallest source–detector separation yields the lowest number of detected photons and therefore represents the worst-case COV, satisfying this criterion at the minimum radius guarantees that all larger separations automatically meet the same or a more stringent precision requirement.

In the second stage, the detected-photon data are used to compute reflectance values according to *Algorithm 2*. A minimal coding example demonstrating the simulation configuration, execution, and reflectance calculation is publicly available at GitHub. *Algorithms 1* and *2* are intended to be used in conjunction with this published code. The full dataset is not publicly available due to its size, but can be obtained from the corresponding author upon reasonable request. For clarity, implementation details such as error handling, bookkeeping (e.g., counters and updates), diagnostics, and fail-safe mechanisms are omitted from this description.

Please note that all simulations were performed using Monte Carlo eXtreme (MCX) through its MATLAB interface, MCXLAB, using MCX v2025.10 ("Jumbo Jolt"). The software was executed within MATLAB R2025a on a Linux workstation running Ubuntu 24.04.2 LTS (Noble Numbat). The scripts provided in the demonstration repository are intended as illustrative examples and will not necessarily be maintained for compatibility with future software updates.

Algorithm 1: Absorption-free Monte Carlo simulations and detected-photon collection

Input: Grids: $\mathcal{G}_{\mu_s'}$ (reduced scattering), $\mathcal{G}_{\mathrm{NA}}$ (numerical apertures), $\mathcal{P}$ (phase functions $p(\theta)$).

Constants: Smallest spot diameter $d_{\min}$; photon threshold for smallest spot $N_{\min}$; maximum fiber diameter $d_{f,\max}$; refractive indices n_{tissue}, n_{ext}; batch size $N_{\mathrm{phot}}^{\mathrm{batch}} = 5 \times 10^7$.

Output: Per-configuration detected-photon data $\mathcal{D} = \{(x, y, \ell)\}$, and total number of launched photons.

Phase 1 — Definitions and initialization

---

foreach NA $\in \mathcal{G}_{\mathrm{NA}}$ **do**

  Compute and store acceptance angle

$$\theta_{\max}(\mathrm{NA}) \leftarrow \arcsin\left(\frac{\mathrm{NA}}{n_{\mathrm{tissue}}}\right).$$

foreach $p(\theta) \in \mathcal{P}$ **do**

  Compute first angular moment for this phase function

$$g[p(\theta)] \leftarrow \int p(\theta) \cos\theta \, \mathrm{d}\Omega.$$

  Construct and store the inverse cumulative distribution function ICDF$[p(\theta)]$ for angular sampling.

  foreach $\mu_s' \in \mathcal{G}_{\mu_s'}$ **do**

    Compute and store the corresponding scattering coefficient

$$\mu_s(\mu_s', p(\theta)) \leftarrow \frac{\mu_s'}{1 - g[p(\theta)]}.$$

Instantiate MCX configurations for all $(\mu_s, \mathrm{NA}, p(\theta))$ as described in Section 2.3, using the corresponding $\theta_{\max}(\mathrm{NA})$, $g[p(\theta)]$, and ICDF$[p(\theta)]$.

Phase 2 — Absorption-free Monte Carlo simulations

---

foreach $(\mu_s, \mathrm{NA}, p(\theta))$ **do**

  Initialize detected-photon list $\mathcal{D} \leftarrow [\,]$.

  Initialize counter for smallest spot: $N_{\mathrm{det}}^{\min} \leftarrow 0$.

  Initialize batch counter: $N_{\mathrm{batch}} \leftarrow 0$.

  while $N_{\mathrm{det}}^{\min} < N_{\min}$ **do**

    Launch $N_{\mathrm{phot}}^{\mathrm{batch}}$ photons and increment batch counter: $N_{\mathrm{batch}} \leftarrow N_{\mathrm{batch}} + 1$.

    foreach *exiting photon* **do**

      Extract exit position (x, y), exit angle θ_{out}, and path length ℓ.

      Compute radial distance $\rho \leftarrow \sqrt{x^2 + y^2}$.

      if $\theta_{\mathrm{out}} \leq \theta_{\max}(\mathrm{NA})$ ***and*** $\rho \leq d_{f,\max}/2$ **then**

        Append (x, y, ℓ) to $\mathcal{D}$.

        if $\rho \leq d_{\min}/2$ **then**

          Increment $N_{\mathrm{det}}^{\min}$.

  Compute total number of launched photons:

$$N_{\mathrm{launched}} \leftarrow N_{\mathrm{batch}} \cdot N_{\mathrm{phot}}^{\mathrm{batch}}.$$

  Save $\mathcal{D}$ and N_{launched} for this configuration.

Algorithm 2: Reflectance calculation using Beer–Lambert reweighting and single-integral approximation

Input: Unique simulation configurations (NA, μ_s', $p(\theta)$); absorption grid $\mathcal{G}_{\mu_a}$; fiber diameters $\mathcal{D}_f$; per-configuration metadata (file paths, launched-photon counts).

Output: Dataset populated with R_{total} for all (NA, μ_s', $p(\theta)$, μ_a, d_f).

Phase 1 — Define ranges and load detected-photon data

Define fiber diameters $\mathcal{D}_f$ and absorption values $\mathcal{G}_{\mu_a}$.
Load file paths for all available (NA, μ_s', $p(\theta)$).
Initialize or expand the dataset to include all required parameter combinations.
(Optional) Initialize a parallel computation pool.

Phase 2 — Pre-computation

---

foreach *unique configuration* (NA, μ_s', $p(\theta)$) **do**
- Load detected-photon data $\mathcal{D} = \{(x, y, \ell)\}$ from disk.
- Compute radial exit distances once: $\rho \leftarrow \sqrt{x^2 + y^2}$.
- Store the corresponding ρ, ℓ, and N_{launched} for reuse in Phase 3.

Phase 3 — Reflectance computation for each μ_a and d_f

---

foreach *dataset entry* (NA, μ_s', $p(\theta)$, μ_a, d_f) (parallelizable) **do**
- Load the precomputed ρ, path lengths ℓ, and normalization constant N_{launched} corresponding to the current (NA, μ_s', $p(\theta)$) configuration.
- Compute absorption weights: $w \leftarrow \exp(-\mu_a \, \ell)$.
- Define radial bin edges $\{\rho_j\}_{j=0}^{M}$ on $[0, d_f]$.
- Bin photons by ρ into annuli $[\rho_{j-1}, \rho_j]$ and accumulate weighted sums $S_j \leftarrow \sum_{\rho \in [\rho_{j-1}, \rho_j]} w$.
- Compute annular areas $A_j \leftarrow \pi(\rho_j^2 - \rho_{j-1}^2)$ and radial reflectance

$$R(\bar{\rho}_j) \leftarrow \frac{S_j}{A_j \, N_{\text{launched}}}, \qquad \bar{\rho}_j = \frac{\rho_{j-1} + \rho_j}{2}.$$

- Evaluate the disk-distance probability density $p(\rho, d_f)$ on the same radial grid.
- Compute total reflectance via the single-integral approximation using numerical integration

$$R_{\text{total}}(\mu_a, d_f) = \frac{\pi}{4} \, d_f^2 \int_0^{d_f} R(\rho) \, p(\rho, d_f) \, \mathrm{d}\rho.$$

- Store R_{total} in the dataset.

.

2.3 Monte Carlo configuration

Geometry and medium

**Domain type**	Homogeneous, semi-infinite slab (photon escape at $z = 0$)
**Physical size**	$100 \times 100 \times 100$ mm^3
**Voxel discretization**	$1 \times 1 \times 1$ voxel
**Voxel size**	100 mm
**Refractive index (medium)**	$n_{\text{tissue}} = 1.35$
**Refractive index (external)**	$n_{\text{ext}} = 1.45$ (SFR) / 1.00 (iSFR)
**Boundary conditions**	Absorbing side and bottom boundaries; top surface includes Fresnel reflection and transmission; specular reflection at the illumination boundary disabled

Optical properties

**Absorption coefficient**	$\mu_a = 0$ during MC simulations (absorption applied via Beer–Lambert reweighting in Algorithm 2)
**Scattering coefficient**	$\mu_s = \mu_s'/(1-g)$, with g the first angular moment of $p(\theta)$
**Phase function**	User-defined $p(\theta)$; inverse-CDF sampler generated per configuration

Source

**Source type**	Pencil beam
**Source position**	$(x, y, z) = (\frac{N_x}{2}, \frac{N_y}{2}, 0)$ at the top surface (grid coordinates)
**Launch direction**	$(0, 0, 1)$, normal to the surface and into the medium
**Photons per batch**	5×10^7
**Angular launch distribution**	Uniform within NA-limited cone, $\theta_{\max} = \arcsin(\text{NA}/n_{\text{tissue}})$
**Random number seed**	`seed` = simulation ID (unique per (NA, μ_s', $p(\theta)$) and batch)
**Execution mode**	GPU acceleration enabled; MCX autopilot active

Detector

**Detection surface**	Entire illumination plane at $z = 0$ (tissue surface)
**Acceptance criterion**	$\theta_{\text{out}} \leq \arcsin(\text{NA}/n_{\text{tissue}})$
**Recorded per photon**	Exit angle θ_{out}, exit position (x, y), path length ℓ
**Radial truncation**	Photons with $\rho > d_{\max}/2$ discarded
**Stored quantities**	Radial exit distance ρ, path length ℓ

2.4 Phase function discretization and p_{sb} calculation

During the discretization of phase functions, we observed numerical instabilities affecting both the calculation of p_{sb} and the resulting reflectance. These instabilities originate from insufficient sampling of the $\cos\theta$ domain, particularly in regions corresponding to strong forward or backward scattering.

Because the definition of p_{sb} involves integration over narrow angular ranges, numerical errors in the backscattering region ($\cos\theta \approx -1$; $179° - 180°$) can propagate strongly. To ensure convergence of the trapezoidal integration of the phase function, we recommend using at least 10^6 sampling points (or alternatively, adaptive quadrature techniques) to mitigate discretization effects. Figure 3A illustrates the resulting p_{sb} values for different $\cos\theta$ discretizations when computing an example TTHG phase function with parameters $\alpha = 0.95$, $g_f = 0.9$, and $g_b = -0.05$, which is strongly forward-peaked.

When computing the reflectance for this same phase function, the analytical probability density function $p(\theta)$ must be converted into its inverse cumulative distribution function (ICDF), which the MCX algorithm internally samples to determine scattering angles. We observed that providing MCX with an insufficiently sampled ICDF leads to noticeable deviations in the simulated reflectance. Figure 3B shows the resulting reflectance values after 5×10^3 Monte Carlo runs for identical soft-tissue–like parameters, when generating the ICDF using different discretizations: 1k, 5k, and 10k points. Because the reflectance is computed in the subdiffuse regime, fine sampling of the specified phase function plays a more critical role than might be expected from diffusion-based considerations. Based on these results, a 10k-point ICDF vector was used in all subsequent simulations. Although the effect of finer discretization may appear modest in this example, as the resulting change in reflectance remains below 1%, it is important to recognize that subdiffuse photon transport is inherently more sensitive to scattering-angle distributions. In contrast to the diffuse regime, where phase-function effects are largely averaged out, small inaccuracies in angular sampling can directly propagate into reflectance errors. Given that inverse optical property retrieval is already an ill-conditioned problem, such modeling-induced errors can accumulate and are therefore best minimized wherever possible. Moreover, as the median model error is on the order of 5%, a discretization-induced error of 1% is not negligible.

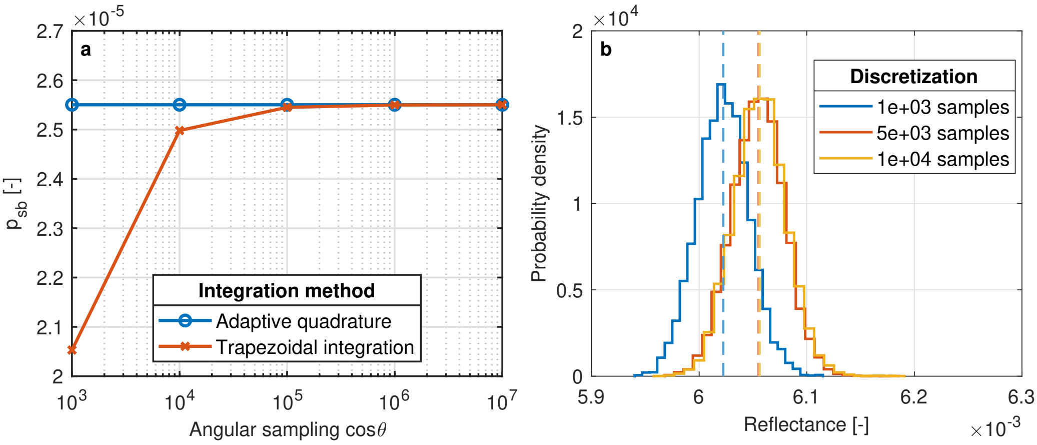

Figure 3: Discretization effects for a TTHG phase function with parameters $\alpha = 0.95$, $g_f = 0.9$, and $g_b = -0.05$. (a) Computed p_{sb} as a function of the number of discretization points used to construct the ICDF, showing convergence toward a stable value beyond a critical sampling density. (b) Propagation of discretization-induced errors into the simulated reflectance, illustrating how undersampling of the phase function, particularly in the $\cos\theta \approx -1$ region, leads to numerical artifacts in the reflectance through inaccurate scattering-angle sampling.

2.5 Stochastic MC noise

Because Monte Carlo simulations are inherently stochastic, a sufficiently large number of photons must be simulated (and detected) to obtain statistically reliable reflectance estimates. To ensure this, our algorithm (Sec. 2.2) implements a stopping criterion that guarantees at least 10^5 detected photons for the smallest fiber diameter in each (μ_s', NA, $p(\theta)$) configuration. For larger fiber diameters, more photons are always detected under identical conditions.

This threshold was established through an analysis across different optical and geometrical regimes. Specifically, we evaluated combinations of $\mu_s' = \{0.5, 10\}$ mm^{-1}, $g = \{0.5, 0.9\}$, $\mu_a = \{1, 10\}$ mm^{-1}, $d_f = \{0.1, 2.0\}$ mm, and NA $= \{0.1, 0.22\}$. For each combination of μ_s', g, and NA, simulations were repeated until a total of 10^{11} photons were launched. The resulting detected-photon arrays were randomly permuted and subdivided into independent subsets containing different numbers of photons. For each subset, reflectance was computed for the various values of μ_a and d_f. The coefficient of variation (COV) was then evaluated as a function of the number of detected photons, defined as COV $= \sigma_R/\langle R\rangle$, where σ_R and $\langle R\rangle$ denote the standard deviation and mean reflectance across the independent subsets. At least 20 statistically independent subsets were used to estimate the COV reliably.

Figure 4 summarizes the results. Each dotted line represents one unique combination of the investigated parameters and is color-coded by $\mu_a \cdot d_f$. The analysis showed that μ_a and d_f had the dominant influence on the number of photons required for convergence. In contrast, variations in μ_s', g, and NA had only a minor effect.

As expected, the COV decreased approximately with the inverse square root of the number of detected photons ($\alpha \cdot 1/\sqrt{N_{\text{det}}}$). Based on these results, a minimum of 10^5 detected photons per configuration was selected as a practical threshold. This ensures that, across all simulated parameter combinations, reflectance was computed with sufficient photons to keep the COV below 1% for all data points used in constructing the model.

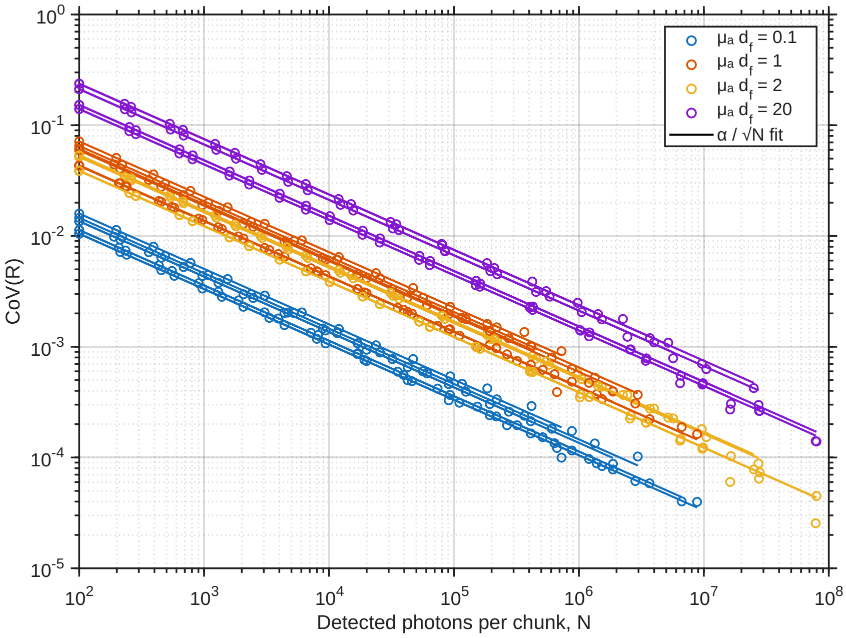

Figure 4: Coefficient of variation of the reflectance as a function of the number of detected photons. For each COV estimate, independent photon subsets were used, with a minimum of 20 batches. For larger detected-photon batches, fewer independent subsets could be formed, resulting in a slightly larger deviation from the fitted trend compared to smaller batches.

3 Results across numerical apertures

All fitted model parameters for both SFR and iSFR configurations are summarized in Table 4 of the main paper. The overall median absolute errors remain consistent across all NAs and among the fitted parameters; only a_2 exhibited a systematic dependence on NA, decreasing with increasing aperture size. This trend, illustrated in Fig. 5, enables interpolation of a_2 to intermediate NA values, thereby extending the model's applicability without requiring additional fitting. All other parameters (a_1, a_3, b_1, b_2) remained effectively invariant across NAs.

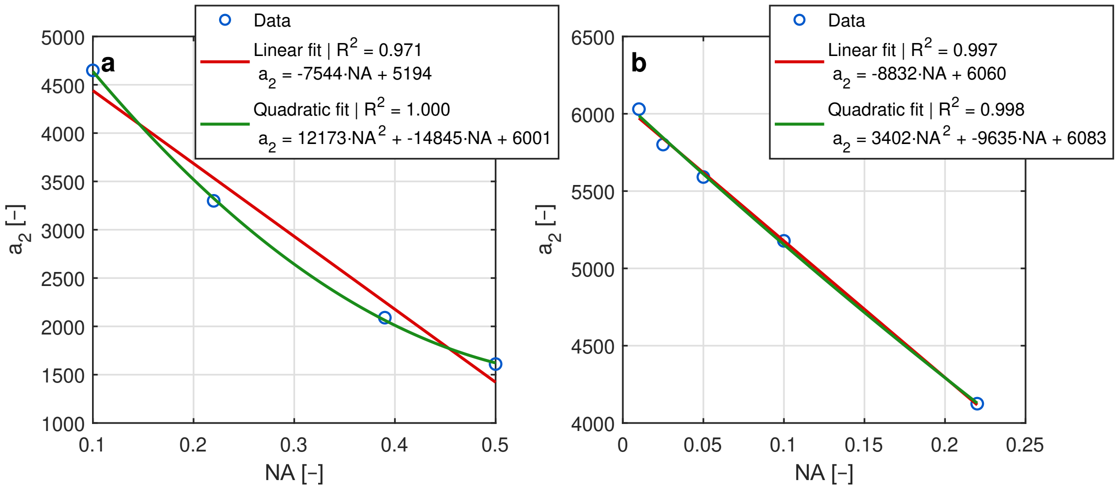

Figure 5: Dependence of the model parameter a_2 on NA. (**a**) SFR and (**b**) iSFR. The fit coefficients and coefficients of determination (R^2) are indicated in the legends.

In theory, one could reformulate X (Eq. 7, main paper) to include an NA dependency that enters through a_2 in the form of a linear or quadratic function. However, we have left out such analysis deliberately due to the sparse sampling of NA and the lack of investigation (and physical motivation) regarding the scaling of NA on the ratio X. More research is needed to validate an NA-dependent model; therefore, for now we advise quadratic interpolation for intermediate a_2 values.

4 Additional scaling relationships

As discussed in the main manuscript, two secondary effects were identified in the reflectance dataset that are not fully captured by the current model formulation. Although these effects were not incorporated in the present version, both because the achieved accuracy was already satisfactory and to avoid unnecessary increases in inversion complexity, they reveal subtle scaling behaviors of light–tissue interactions in the subdiffuse regime.

Residual subdiffuse component and deviations from ideal power-law scaling. The absorption-free power-law formulation $X_0 = a_2 \left(\frac{p_{sb}}{(\mu_s' d_f)^2}\right)^{a_3}$ implicitly assumes that, for large $\mu_s' d_f$, the reflectance becomes fully diffuse, such that $X \to 0$. In practice, however, a small residual subdiffuse contribution remains, whose magnitude depends weakly on the phase function. As shown in Fig. 6a, the diffusion-theory prediction (black line) converges toward a single asymptotic limit, whereas absorption-free Monte Carlo simulations for three different phase functions plateau at slightly different reflectance levels for large $\mu_s' d_f$. This residual offset varies with the detailed angular structure of the phase function, as suggested by its dependence on p_{sb}. Because diffusion theory does not account for such phase-function dependence, the ratio X does not strictly converge to zero.

When considering the full set of simulated (absorption-free) X_0 values across all combinations of (μ_s', d_f, p_{sb}), Fig. 6b shows that this behavior manifests as a gradual deviation from the fitted power-law trend in the highly diffuse regime, corresponding to low X_0. Here, X_0 is obtained by subtracting the diffusion-theory prediction (R_{dif}) from the total reflectance computed by the Monte Carlo simulations, thereby isolating the semi-ballistic reflectance contribution used to define X_0. Figure 6b further confirms that the transition toward the diffuse limit is not perfectly described by a single power-law dependence. While an extended formulation that combines a power-law term with a finite plateau could, in principle, capture this behavior more accurately, such refinements were not pursued in the present work.

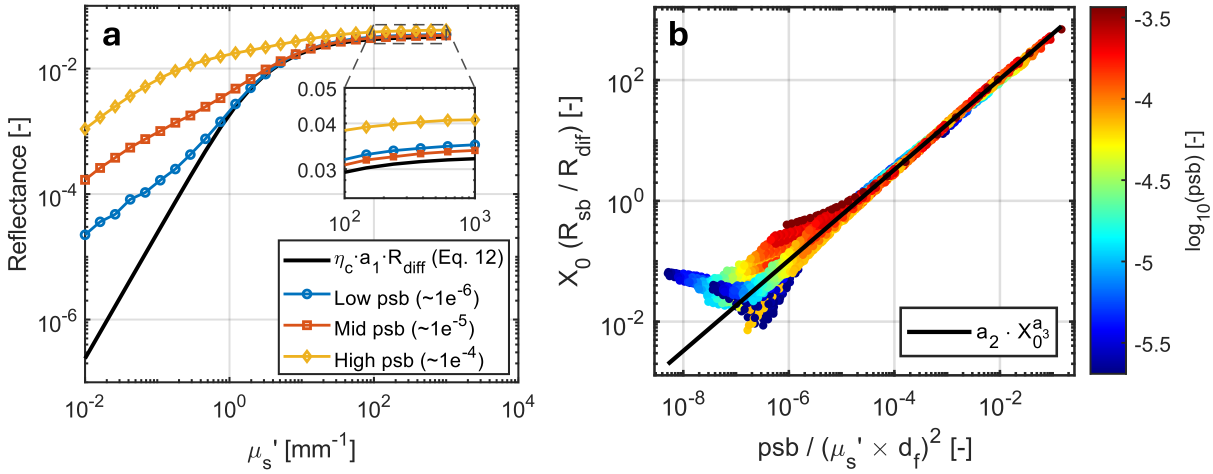

Figure 6: (a) Absorption-free reflectance predicted by diffusion theory (black line) compared to Monte Carlo simulations for three phase functions, showing that for large $\mu_s' d_f$ the total reflectance R_{tot} plateaus slightly above the diffuse prediction R_{dif}, with the offset depending on the input phase function. (b) Simulated values of X for all combinations of (μ_s', d_f, p_{sb}), illustrating increasing deviation from the fitted power-law trend in the highly diffuse regime ($X \to 0$).

Absorption correction

Following Post *et al.*, absorption was modeled using a multiplicative correction applied to the absorption-free quantity X_0, such that $X_{\mu_a} = X_0 \exp\big[b_1(\mu_a/\mu_s')^{b_2}\big]$, or equivalently, $X_{\mu_a}/X_0 = \exp\big[b_1(\mu_a/\mu_s')^{b_2}\big]$. While this exponential relation captures the overall absorption dependence, there appears to be an additional dependence on $p_{sb}/(\mu_s' d_f)^2$. Figure 7 shows the ratio X/X_0 as a function of μ_a/μ_s', color-coded by $p_{sb}/(\mu_s' d_f)^2$. The exponential correction deviates as $X \to 0$, which is consistent with the presence of the residual subdiffuse component discussed above. The systematic additional scaling with $p_{sb}/(\mu_s' d_f)^2$ indicates that a single exponential dependence on μ_a/μ_s' does not fully capture the influence of absorption. Although extending the model to incorporate this additional dependency could further improve accuracy, such refinements were not explored here.

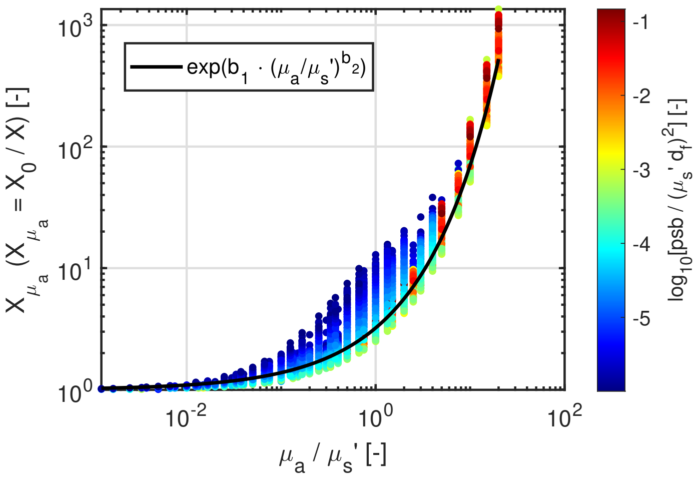

Figure 7: Absorption correction ratio $X/X_0 = \exp[b_1(\mu_a/\mu_s')^{b_2}]$ as a function of μ_a/μ_s', color-coded by $p_{sb}/(\mu_s' d_f)^2$. Because $X_0 = f\big(p_{sb}/(\mu_s' d_f)^2\big)$ the figure illustrates that the absorption correction exhibits an additional dependence on X_0.

5 Inversion routine

To retrieve the optical-property parameterizations from the simulated reflectance spectra, we implemented a nonlinear inverse solver based on a multistart Levenberg–Marquardt optimization. For each inversion, randomized initial guesses were drawn uniformly within physically meaningful parameter bounds (Table 1), after which each candidate solution was refined using MATLAB's `lsqnonlin` routine. The optimization minimizes a relative least-squares cost function,

$$\chi^2(\boldsymbol{\theta}) = \sum_{\lambda} \left(\frac{R_{\text{model}}(\lambda; \boldsymbol{\theta}) - R_{\text{simulation}}(\lambda)}{R_{\text{simulation}}(\lambda)} \right)^2,$$

where $\boldsymbol{\theta}$ denotes the parameter vector, $R_{\text{model}}(\lambda; \boldsymbol{\theta})$ is the modeled reflectance spectrum, and $R_{\text{simulation}}(\lambda)$ is the simulated reference spectrum. Residuals are inversely weighted by the absolute reflectance to prevent spectral regions with high reflectance amplitudes from dominating the fit, thereby promoting balanced sensitivity across the wavelength range.

Each inversion estimates the parameter vector $\boldsymbol{\theta} = [a, b, v_{f,\text{blood}}, sO_2, p_1, p_2, p_3]$,where a and b parameterize the amplitude and spectral slope of the reduced scattering coefficient $\mu_s'(\lambda)$. The blood volume fraction $v_{f,\text{blood}}$ and oxygen saturation sO_2 determine the absorption coefficient $\mu_a(\lambda)$, which is modeled as

$$\mu_a(\lambda) = v_{f,\text{blood}} \left[sO_2\, \mu_{a,\text{oxy}}(\lambda) + (1 - sO_2)\, \mu_{a,\text{deoxy}}(\lambda) \right].$$

The wavelength dependence of the phase-function parameter $p_{sb}(\lambda)$ is described by a third-order polynomial,

$$p_{sb}(\lambda) = 10^{-5} \left[p_1 \left(\frac{\lambda}{650\text{ nm}} \right) + p_2 \left(\frac{\lambda}{650\text{ nm}} \right)^2 + p_3 \left(\frac{\lambda}{650\text{ nm}} \right)^3 \right],$$

where the prefactor 10^{-5} rescales the polynomial coefficients and improves numerical conditioning during optimization.

Each spectrum was fitted independently using fifty randomized starting points, executed in parallel through MATLAB's `MultiStart` interface. The solution yielding the lowest residual norm was retained for subsequent analysis. All inversions reported in this work were performed using a fixed numerical aperture of NA = 0.05 and a constant $A = 2.6355$ in the diffusion equation, corresponding to the refractive index mismatch between air and tissue. Optimization tolerances were set to 10^{-16} to prevent premature termination. Although this choice increased computational runtime moderately, it ensured consistent convergence across all fits.

Table 1: Parameter bounds used in the multistart Levenberg–Marquardt inversion.

**Parameter**	**Symbol**	**Lower bound**	**Upper bound**
Scattering amplitude	a	0	50
Scattering slope	b	0	5
Blood volume fraction	$v_{f,\text{blood}}$	0	0.2
Oxygen saturation	sO_2	0	1
Phase-function coefficients	p_1, p_2, p_3	-10^3	10^3

For the demonstrations presented here, no additional normalization or regularization terms were included. This choice was made to maintain transparency in how each parameter contributes to the fit. As a result, the inversion exhibits unbalanced parameter sensitivities and noticeable competition, most prominently between μ_s' and p_{sb}. Future implementations could mitigate these effects through parameter normalization, adaptive weighting, or regularization strategies designed to stabilize correlated parameters while preserving physical interpretability.

References

[1] Post, A. L., Sterenborg, H. J. C. M., Woltjer, F. G., van Leeuwen, T. G. & Faber, D. J. Subdiffuse scattering model for single fiber reflectance spectroscopy. *J. Biomed. Opt.* **25**, 015001 (2020).

[2] Faber, D. J., Post, A. L., Sterenborg, H. J. C. M. & van Leeuwen, T. G. Analytical model for diffuse reflectance in single fiber reflectance spectroscopy. *Opt. Lett.* **45**, 2078–2081 (2020).

[3] García-Pelayo, R. Distribution of distance in the spheroid. *J. Phys. A: Math. Gen.* **38**, 3475–3482 (2005).
